# Bromelain Restores Glutamatergic Homeostasis via Regulation of NR2A, GLT-1, EAAC1, and xCT in Arsenic-Induced Cerebral Cortex and Hippocampal Neurotoxicity

**DOI:** 10.12688/f1000research.169308.1

**Published:** 2025-10-07

**Authors:** Anyanwu Emeka Godson, Kpokuechukwu Chinua Ogonnadi, Augustine Uchenna Agu, Nto Johnson Nto, Ikechukwu Aniaku, Yadilichi Yvonne Nwabueze, Vivian Onyinye Ojiakor, Anyanwu Chinyere Nkemjika

**Affiliations:** 1 Department of Anatomy, Faculty of Biomedical Sciences, Kampala International University - Western Campus, Bushenyi, Western Region, Uganda; 2 Department of Anatomy, Faculty of Basic Medical Sciences, Godfrey Okoye University, Enugu, Enugu, Nigeria; 3 Department of Anatomy, Faculty of Basic Medical Sciences, University of Nigeria Faculty of Medical Sciences, Nsukka, Enugu, Nigeria; 4 Department of Anatomy, Faculty of Basic Medical Sciences, University of Nigeria Faculty of Medical Sciences, Nsukka, Enugu, Nigeria; 5 Department of Anatomy, Faculty of Basic Medical Sciences, University of Nigeria Faculty of Medical Sciences, Nsukka, Enugu, Nigeria; 6 Department of Anatomy, Faculty of Basic Medical Sciences, University of Nigeria Faculty of Medical Sciences, Nsukka, Enugu, Nigeria; 7 Department of Anatomy, Faculty of Biomedical Sciences, Kampala International University - Western Campus, Bushenyi, Western Region, Uganda; 8 Department of Microbiology and Immunology, Faculty of Biomedical Sciences, Kampala International University - Western Campus, Bushenyi, Western Region, Uganda

**Keywords:** Neuroprotection, Excitotoxicity, Cognitive dysfunction, Pineapple-derived protease, Synaptic plasticity

## Abstract

**Background:**

Chronic arsenic exposure interferes with hippocampal-dependent cognition through glutamate excitotoxicity, which in turn interferes with the regulation of receptors and transporters. Bromelain, a combination of proteolytic enzymes derived from Ananas comosus, is known to have neuroprotective effects; however, the mechanisms by which it counteracts glutamate-mediated toxicity in the brain are poorly understood. This study investigated the potential of bromelain to normalize glutamatergic homeostasis and cognitive function in arsenic-treated rats by specifically examining the NMDA receptor subunit NR2A and glutamate transporters GLT-1, EAAC1, and xCT.

**Methods:**

Seventy-two adult male Wistar rats (200-220 g) were randomly divided into nine groups (n=8 each): control, arsenic-only (20 mg/kg/day sodium arsenite, administered via oral gavage for 14 days), bromelain-only (5, 10, or 15 mg/kg/day, oral gavage), arsenic plus bromelain (at the same doses), and arsenic plus donepezil (2 mg/kg/day, intraperitoneal). Arsenic was coadministered with bromelain and donepezil for 14 consecutive days. The Morris water maze test was used to assess the cognitive performance. Glutamate concentration was measured using sandwich Enzyme-Linked Immunosorbent Assay [ELISA] and total RNA was isolated to perform RT-qPCR to evaluate the expression of NR2A, GLT-1, EAAC1, and xCT.

**Results:**

Exposure to arsenic impaired spatial memory, increased glutamate levels, and downregulated the expression of NR2A and transporter genes. These effects were reversed by co-treatment with bromelain, especially at 10 mg/kg, which re-established gene expression and lowered glutamate levels. Bromelain at 15 mg/kg was more effective than donepezil in improving glutamate clearance and cognitive performance.

**Conclusions:**

Bromelain provides multi-target neuroprotection in arsenic neurotoxicity, rescuing glutamatergic homeostasis by coordinated upregulation of NMDA receptor NR2A and the transport network (GLT-1, EAAC1, xCT), accompanied by spatial learning and memory improvement. The demonstrated dose-response, dosing-related superiority over 15 mg/kg donepezil, and lack of overt adverse effects support its translational potential as a safe, plant-based adjunct to excitotoxic diseases and toxin-exposed populations.

## Introduction

Memory, learning, and decision making are basic aspects of daily life, and their impairments are features of neurodegenerative and psychiatric disorders. Diseases, such as Alzheimer’s disease and schizophrenia, are associated with deterioration in cognitive performance. These disorders are strongly associated with disorders of excitatory neurotransmission, specifically N-methyl-D-aspartate receptors (NMDARs). ^1– 4^ NMDARs are ion channels which respond to glutamate and have a major role in establishing synaptic plasticity as well as memory. Marginal alterations in the functionality of NMDARs may exert lethal effects on neural circuitry. ^5– 7^ Overactivation of NMDARs, for example, calcium overload, results in excitotoxic pathways that cause neuronal damage, whereas hypoactivation of NMDAR has been linked to cognitive impairments in disorders such as schizophrenia and age-related decline. ^8, 9^ The integrity of cognition depends therefore on the maintenance of adequate functions of elements of NMDAR and their expression.

In addition to receptors, glutamate transporters are also important in the regulation of excitatory signaling. ^10, 11^ Astrocytes and neurons have excitatory amino acid transporters (e.g. GLT1/EAAT2 in astrocytes and EAAC1/EAAT3 in neurons) that remove glutamate quickly out of synapses, thereby halting neurotransmission and any accumulation of exogenous glutamate that is toxic. ^12– 14^ Glutamate homeostasis is also mediated by the cystine glutamate antiporter (system x-c (containing the xCT subunit)) linking extracellular cystine availability with intracellular glutamate release. ^15^ Dysregulation of these transporters aggravates neuronal dysfunction; interference in stimulation of glutamate transport causes extracellular glutamate overflow, excessive activation of NMDARs, oxidative stress, and subsequent neurotoxicity. ^7^ For example, loss of astrocytic GLT1 leads to early memory and learning deficits, ^16^ whereas loss of neuronal GLT1 leads to memory deficits later in life. ^17, 18^ Similarly, reduced levels or functions of GLT1 have been correlated with cognitive impairment in the context of Alzheimer, Parkinson and ALS. ^16^ Neurodegeneration is caused by glutamate excitotoxicity, transporter failure, and NMDAR overstimulation. Based on these observations, the development of therapies that improve the clearance of glutamate by upregulating transporters (in particular astrocytic EAAT2/GLT1) has proven to be an attractive approach to prevent excitotoxic damage in the brain. ^4, 8^ In fact, it is possible to reverse cognitive deficit in animal model with pharmacological up-regulation of GLT1 and the GLT1-up-regulating drugs such as ceftriaxone can enhance cognition in animals models of Alzheimer disease. ^19, 20^

Previous studies have demonstrated the neuroprotective effects and underlying mechanisms of several dietary phytochemicals, including fisetin, ^21, 22^ zingerone, ^23– 26^ morin, ^27^ chrysin, ^28^ and curcumin. ^29, 30^ Among these bioactive compounds, bromelain, a multi-component protease extracted from the stems and fruits of Ananas comosus (pineapple), has also attracted interest because of its traditional anti-edematous and anti-inflammatory properties. ^31– 33^ New evidence suggests that bromelain has neuroprotective effects too. ^34, 35^ It has the ability to regulate both oxidative stress and neuroinflammatory models, resulting in decreasing neuronal death and improving paths in models of neurological disease. Bromelain has demonstrated effectiveness in reducing cognitive decline and disease-related pathology in experimental rodent models of Alzheimer’s and Parkinson’s disease. ^36– 38^ Bromelain enhanced spatial learning and memory in a mouse model of Alzheimer without any side effect, and it was proposed to produce its effect through anti-oxidative effects. In another sciatic nerve injury model, bromelain decreased pro-inflammatory mediators, which correlated with decreased pain and anxiety behaviors. Despite these encouraging results, the effects of bromelain on neurotransmitter systems, especially glutamate transport and NMDAR-mediated signaling, are not fully understood.

Arsenic neurotoxicity is a clinically relevant model for excitotoxic cognitive loss. ^39^ Arsenic (As) is a common environmental pollutant that has easy access to the blood-brain barrier and is deposited in the hippocampus and other areas. Cognitive and behavioral disorders in humans have been documented following chronic (and even low) exposure to arsenic, and this problem is acknowledged as a predisposing factor for neurological disorders. ^40, 41^ Arsenic, at the cellular level, arsenic triggers a range of detrimental mechanisms, such as oxidative stress, mitochondrial dysfunction, and inflammation, which culminate in interfering with neuronal activity. It is noteworthy that neurotoxicity associated with arsenic is due to the disruption of glutamate homoeostasis; it is either there is an excess of extracellular glutamate and, consequently, an overactivation of NMDARs, resulting in impairment of synaptic plasticity and death of neurons. Earlier exposure of rodents to arsenic leads to downregulation of glutamate transporter expression (GLT1, EAAC1, xCT) and NMDAR subunits, and has been linked to memory problems. ^41– 44^ Such changes are a reflections of excitotoxic processes and indicate that cognitive dysfunction as a result of arsenic exposure is at least in some part a result of glutamatergic dysregulation.

Therefore, we hypothesized that bromelain may reverse arsenic-mediated cognitive deficits through its ability to normalize excitatory neurotransmission. In particular, we aimed to establish whether treatment with bromelain increases the expression of the main glutamate transporters (GLT1, EAAC1, and xCT) and the NMDAR subunit NR2A in the hippocampus and prefrontal cortex, thus decreasing the level of glutamate excitotoxicity and enhancing cognitive performance. We aimed to reduce excitotoxic brain damage by the mechanistic action of bromelain on these molecular targets using a rat model of arsenite exposure. This paper provides an idea of how bromelain can help maintain synaptic and cognitive health against the pressure of toxic environmental stresses.

## Methods

### Ethical approval

The experimental procedure was approved by the Institutional Animal Care and Use Committee of the College of Medicine, University of Nigeria Institutional Animal Care and Use Committee (IACUC) (ethics approval no. COMHREC/2025/01/025), and all efforts were made to avoid unnecessary suffering in animals. Experiments were performed under the guidelines of the U.S. National Research Council Guide for the Care and Use of Laboratory Animals and ARRIVE 2.0 guidelines.

### Experimental animals and housing conditions

Eight-week-old Male Wistar rats (Rattus norvegicus) weighing 200-230 g were procured from the animal facility of the University of Nigeria. The rats were kept in a room with controlled temperature (28°C) and humidity (50–60°C) and 12 h/12 h light/dark cycle (8 rats per cage). Animals were allowed free food intake of standard chow and water. The acclimatization period to the facility was 14 days after the start of the experiment.

### Sample size and power calculation

The sample size was calculated a priori to achieve sufficient statistical power to identify biologically relevant differences between experimental groups using the G*Power software (version 3.1.9.7, Universitat Dusseldorf, Germany). One-way analysis of variance ANOVA (fixed effects, omnibus, one factor) was selected because the primary comparisons involved multiple independent treatment groups (nine of them). The parameters used were based on the reported effect sizes in other rodent neurotoxicity studies that measured the change in glutamate transporter and receptor expression after pharmacological manipulation (Cohen’s f = 0.5, large effect size).

• **α** (Type I error probability): 0.05 (two-tailed)
• **Power (1–β)**: 0.80
• **Number of groups**: 9
• **Effect size (Cohen’s *f* )**: 0.5

The analysis showed a minimum of eight animals per group to attain the desired statistical power. This figure is adequate to maintain the balance between scientific sensitivity and ethical minimization of the use of animals in line with the 3Rs principle (Replacement, Reduction, Refinement).

Eight adult male Wistar rats were used in each group, with a total of 72 rats in the nine treatment groups. This sample size is sufficient to provide strong power to detect treatment effects on biochemical and molecular responses (NR2A, GLT-1, EAAC1, and xCT expression) and a reasonable amount of attrition due to unanticipated health problems.

### Experimental grouping and treatment regimen

The 72 rats were randomly divided into nine experimental groups (n = 8 per group) following acclimatization as follows:

• **Group A**: Normal Control: no toxin or drug exposure (vehicle only; standard feed and water).
• **Group B**: Arsenic Control: Sodium arsenite (20 mg/kg body weight, p.o.) for 14 days to induce cognitive impairment.
• **Group C**: Bromelain Low: Bromelain (5 mg/kg body weight, p.o.) for 14 days.
• **Group D**: Bromelain Medium: Bromelain (10 mg/kg, p.o.) for 14 days.
• **Group E**: Bromelain High: Bromelain (15 mg/kg, p.o.) for 14 days.
• **Group F**: Arsenic + Bromelain Low: Sodium arsenite (20 mg/kg, p.o., 14 days), followed by bromelain (5 mg/kg, p.o., 14 days).
• **Group G**: Arsenic + Bromelain Medium: Sodium arsenite (as above) followed by bromelain (10 mg/kg, p.o., 14 days).
• **Group H**: Arsenic + Bromelain High: Sodium arsenite (14 days/20 mg/kg) followed by bromelain (14 days/15 mg/kg).
• **Group I**: Arsenic + Donepezil (Positive Control): sodium arsenite (20 mg/kg, 14 days) followed by donepezil (2 mg/kg, p.o., 14 days).

The dosage regimen was based on previous studies on arsenic neurotoxicity and bromelain efficacy. ^37, 43^ In the first two weeks, sodium arsenite (NaAsO 2; Merck KGaA, Darmstadt, Germany) was administered by oral gavage once daily. The 20 mg/kg dose was selected from pilot studies as the lowest dose that induced significant learning memory deficits without excessive systemic toxicity.

Freshly prepared in distilled water, bromelain (Pure Encapsulations 1000 mg, Sudbury, MA, USA) was orally gavaged daily to the respective groups in the second two weeks (Days 15-28). The doses (5, 10, 15 mg/kg) were selected to encompass a low-to-high therapeutic range based on previous animal investigations in which cognitive enhancement was observed at approximately 10 mg/kg. Donepezil (Aricept 2 mg/kg) was used as a reference neuroprotective agent, which has been shown to improve memory in toxin-induced models.

The control and arsenic-only groups were treated with a similar amount of vehicle (distilled water) throughout the treatment period.

### Animal monitoring and welfare

The health status of the rats and their signs of toxicity were monitored daily. Body weight was recorded weekly. No mortalities or major adverse events occurred during the course of the study.

To minimize possible confounding factors, the experiment was conducted in two cohorts, where the representation of the groups was equal. An observer blinded to the group assignments was administered the behavioral testing.

### Experimental timeline

Cognitive performance was assessed after arsenic or control treatment (day 15) using the Morris water maze). Bromelain or donepezil treatments were then applied for the next 14 days. Behavioral testing was performed at the endpoint (day 28) prior to euthanasia and tissue collection.

The arsenic-only group (Group B) was humanely euthanized on day 15 after completing arsenic exposure to obtain maximum arsenic effects without recovery. The remaining rats were euthanized on day 28. The staggered endpoint was intended to distinguish between the acute effects of arsenic and the post-treatment outcomes. Samples were collected at the respective sacrifice times and analyzed behaviorally and biochemically.

**Behavioral assessment: Morris Water Maze Test**

The Morris water maze (MWM) method was used to assess spatial learning and memory before the treatments on the 14th study day and then on study day 28 to measure cognitive impairment and recovery. The MWM instrument was a round pool (126 cm diameter and 75 cm height) that contained water (depth 40 cm) controlled at 24 1 C. Water was opacified with non-toxic opaque paint (white tempera) or skim milk powder to conceal a sunken escape platform. Considering the concept of small areas, four quadrants (NE, NW, SE, and SW) were depicted on the walls of the pool and were located visually using specific markings. During training and testing a clear plastic platform (10 cm diameter) was positioned 2 cm beneath the water surface in the target quadrant (SW quadrant, ~10 cm from the wall).

All the rats underwent two stages acquisition (training) and retrieval (testing). The rats were subjected to four training trials three times during the acquisition phase (days 12-14 of the experiment). During each trial, the rat was calmly placed in the water at a pseudo-random start position (switching between NE, NW, and SE start locations) and given a maximum of 60 s to discover the hidden platform. If a rat was unable to locate within 60 s, it was directed to the platform by the experimenter. The duration was 15 seconds, after which the rats were removed from the platform. This training was modified based on Forero et al. (2023) ^45^ with slight changes. The aim was to allow rats to learn where the hidden platform was using spatial cues.

On the test day, 24 h after the final training trial, a probe trial was conducted (day 15 for the arsenic-only group and day 28 for all others). During the probe test, the platform was removed from the pool. Each rat was dropped into a new starting point and permitted to swim freely all over the arena for 60 s. As the main indicator of spatial memory, escape latency (the time taken to reach the old platform position) was registered. A smaller escape latency indicates an enhanced memory of the location of the platform. Patterns of swim paths were also examined, so that no significant sensorimotor impairments were present (all rats could swim normally). It was filmed using a video camera above the pool, and tracking was conducted manually using a stopwatch (computer automated tracking software was an option, but resource issues were not considered).

**Tissue collection and processing**

At the end of the experiment, the rats were anesthetized (using intraperitoneal injection of sodium pentobarbital (100 mg/kg) followed by decapitation, as recommended by the AVMA Guidelines for the Euthanasia of Animals (2020). This method ensures rapid loss of consciousness and is approved under the COMHREC/2025/01/025 protocol. In the arsenic-only group (Group B), euthanasia occurred on day 15, immediately after the final memory test, whereas in all other groups, the process took place on the 28th day. The schedule ensured that the arsenic-alone animals were tested at the peak of impairment, whereas the treatment groups were examined after the recovery/treatment phase.

Immediately after sacrifice, sterile scissors were used to quickly open the skull. The entire brain was removed and washed with ice-cold saline to remove the blood. To maintain RNA and protein integrity in brain tissues, they were processed on a cold surface. The brains were blotted dry and then meticulously dissected to separate the hippocampus and prefrontal cortex on both sides. The protocol for guided freehand dissection was presented by Spijker. ^46^ Briefly, the brain was longitudinally cut in the midsagittal plane, the hippocampus of each half-brain was pulled out of the latter on the ventral surface and peeled off, and a mild slice was removed from the prefrontal cortex (~3 mm in front of the frontal pole). This dissection procedure produces tissue samples that have highly represented targeted areas, as well as limited cross-contamination between other regions.

The snipped tissues were immediately placed in RNAlater stabilization solution (Ambion, USA) to inhibit RNA degradation. The samples were stored overnight in RNAlater at 4°C followed by storage at -80°C until molecular analysis. (In preliminary tests, a 2-week fixation in RNAlater did not significantly degrade RNA quality; tissues remained suitable for PCR) Part of each hippocampus was also flash-frozen and stored in a freezer at -80°C in order to perform glutamate assay. For this part of our study, we did not subject the animals to perfusion fixation, because our endpoints were molecular. Every effort was made to ensure that animal suffering and pain was brought to the barest minimum.

**RT-qPCR Gene analysis expression**

Total RNA was extracted from the hippocampal and prefrontal cortex using a commercial RNA isolation kit (TransGen Biotech, Beijing, China) according to the manufacturer’s instructions. Approximately 30 mg of tissue was homogenized in 1 mL of TRIzol reagent in the presence of 0.1 M 8-mercaptoethanol (Life Technologies, Gaithersburg, MD, USA) as a reducing agent. The homogenates were maintained at room temperature and incubated for 5 min to ensure that all nucleoprotein complexes were dissociated. Chloroform (200 μL per mL TRIzol) was added, and the samples were shaken vigorously for 15 s and then centrifuged at 12,000 × *g* for 15 min at 4°C. The RNA in the aqueous phase was carefully transferred to a new tube, and an equal amount of 70 percent ethanol was added. A silica gel membrane spin column was used to apply the solution. The column that was bound was washed with the supplied buffers and RNA was eluted with RNase-free water. This step was followed by on-column DNase I digestion step that was meant to avoid contamination with genomic DNA. The quality and quantity of RNA were determined based on absorbance measured at 260 nm and 280 nm using the NanoDrop 2000 (ThermoFisher Scientific), with A260/280 between 1.9 and 2.1. DNA integrity was validated through electrophoresis on a 1 percent agarose gel with clear 18 S and 28 S rRNA bands.

**cDNA Synthesis**

Complementary DNA (cDNA) was synthesized from 2–3 μg of total RNA per sample using a high-capacity reverse transcription kit (Applied Biosystems, Foster City, CA, USA). RNA was initially denatured at 65°C for 5 min and cooled rapidly. The reverse transcription reaction (volume of 20 μL) consisted of RNA template, 4 μL volume of 5x RT buffer, 2 μL of dNTP mix (10 mM each), 1 μL of random hexamer primers, 1ul of Moloney murine leukemia virus (MuLV) reverse transcriptase, and RNase free water. The reaction was incubated at 42°C to be incubated with 30 min after which it was incubated at 95°C to destroy the enzyme after 5 min. The obtained cDNA was kept at -20°C until qPCR.

Real-time PCR: Q-PCR was performed using Rotor-Gene Q real-time PCR with SYBR Green chemistry. The target genes, including cystine/glutamate antiporter xCT (Slc7a11), EAAT1 (Slc1a3), GLT1 (Slc1a2), NMDA receptor NR2A subunit (Grin2a), glial fibrillary acidic protein GFAP (Gfap), and housekeeping gene β-actin (Actb) were prepared using specific primer pairs.

Primer sequences are as follows:

• GFAP (Gfap): Forward 5′-AAAGACACTGAAACAGGAGAGAG-3′, Reverse 5′-GGACTGAGCAACCAGGAATAG-3′
• xCT (Slc7a11): Forward 5′-ACCAACATGGCTGTCACTTAT-3′, Reverse 5′-CTCTGTCTCTGTCTCTGTCTCT-3′
• EAAT1 (Slc1a3): Forward 5′-AAAGAGCAGAGGCGGAATAG-3′, Reverse 5′-AGAAGAAGGCCAAGGTTCAG-3′
• GLT1 (Slc1a2): Forward 5′-ACTGGCTGCTGGATAGAATG-3′, Reverse 5′-CTCGGACTTGGAAAGGTGATAG-3′
• NR2A (Grin2a): Forward 5′-GTCCAACCCTAACACAGTAGAG-3′, Reverse 5′-GTTTAGAGAATCCTGGCGTAGAG-3′
• β-Actin: Forward 5′-ACAGGATGCAGAAGGAGATTAC-3′, Reverse 5′-ACAGTGAGGCCAGGATAGA-3′

Each PCR assay consisted of a 25 μL reaction containing 12.5 μL of 2x SYBR Green Master Mix (Applied Biosystems), 0.5 μM of forward and reverse primer and 2 μL of diluted cDNA template(~100 ng RNA equivalent). The thermal cycling conditions consisted of an initial denaturation at 95°C for 10 min and 35 cycles of 95°C for 30 s, 57–60°C for 40 s and 72°C for 40 s, depending on the primer annealing temperature. An analysis of the melting curve between 60°C and 95°C at cycling completion was also performed to confirm the specificity of amplification (a single product peak per product). Each primer pair contained no-template control (NTC) to rule out possible contamination or primer dimers; the NTCs were negative in each case.

**Quantification**

Cycle threshold (CT) values were obtained for each sample. The expression levels of the target genes were normalized to β-actin as an internal reference in the same sample (ΔCT = CT (target) – CT(β-actin)). The relative expression of each target gene in the treated groups was calculated using the 2–ΔΔCT method, with the normal control Group A (Group A) as the calibrator. This yielded a fold-change in mRNA expression relative to that in the control. Each PCR was run in duplicate, and the average CT was used for analysis. Data are presented as mean fold-change ± standard error (SE) for n = 5 animals per group (except for the arsenic-only group n = 5 at its time point).

**Determination of glutamate level**

The concentration of brain glutamate was determined using a competitive Enzyme-Linked Immunosorbent Assay (ELISA) kit (Analytik Jena GmbH, Germany) according to the manufacturer’s instructions. Hippocampal tissue samples (~50 mg) were homogenized in ice-cold 0.1 M phosphate-buffered saline (pH 7.4) and centrifuged (10,000 g, 10 min, 4°C) to obtain supernatants. The supernatants were discarded, and the samples were diluted and subjected to ELISA.

The principle of the assay is competitive binding; unused glutamate in the samples competes with a glutamate-enzyme conjugate to combine with glutamate-specific antibodies mounted on the wells of a microplate. The wells of a microtiter plate coated with anti-glutamate antibody were filled with 25 μL of each sample or glutamate standard solution. Then, 50 μL of glutamate-type analog conjugated enzyme was added to each well. Adhesive film was applied on the plates and incubated overnight at 4°C with competition binding. The wells were then washed thrice using the wash buffer provided in the kit to remove unbound material. Next, 100 μL of each well was subjected to TMB substrate solution and incubated at room temperature (20-25) for approximately 20 min under gentle shaking in the dark. The reaction was stopped by adding 100 μL of stop solution (1 N HCl), which yielded a yellow color. A microplate reader was used to measure the absorbance (450 nm) with reference absorbance (600 nm).

A standard curve was developed using known glutamate concentrations (ranging from 0 to 50 μg/mL of glutamate), and sample concentrations of glutamate were calculated using the obtained standard curve. All samples were analyzed in triplicate. In this competitive ELISA, the optical density is proportional to the inverse of the glutamate concentration (glutamate in the sample competes with conjugation of the enzyme); the lower the glutamate, the higher the optical density. We generated a standard inhibition plot and calculated the levels of glutamate in each sample, measured in μmol per gram of tissue.

### Statistical analysis

Results are expressed as the mean ± standard error of the mean (SEM). One-way analysis of variance (ANOVA) using the appropriate multiple comparison tests was performed using behavioral (escape latency) and biochemical data (glutamate level and gene expression fold-changes). In particular, all treatment groups were compared with the arsenic-only group using Dunnett’s test, and multiple bromelain dosages and donepezil were compared using Tukey’s HSD test. To examine learning curves in the water maze escape latency (acquisition trial interval over days), two-way repeated-measures ANOVA (factors: group and training day) was used, and Bonferroni correction of multiple comparisons was obtained on each day. All tests were defined at a significance level of p < 0.05. Data were analyzed using GraphPad Prism 8.0 (GraphPad Software, CA, USA). The sample sizes (n = 5) within each group were selected using power calculations against pilot data, where n = 5 was estimated to be able to detect a 20 per cent reduction in escape latency or a 2-fold degree of change in gene expression in a study with an alpha value of 0.05, with a level of power greater than 80 per cent. To prevent bias, all analyses were performed with the experimenter not knowing who was in the group.

## Results

### Behavioral performance in Morris Water Maze

**Figure 1.**
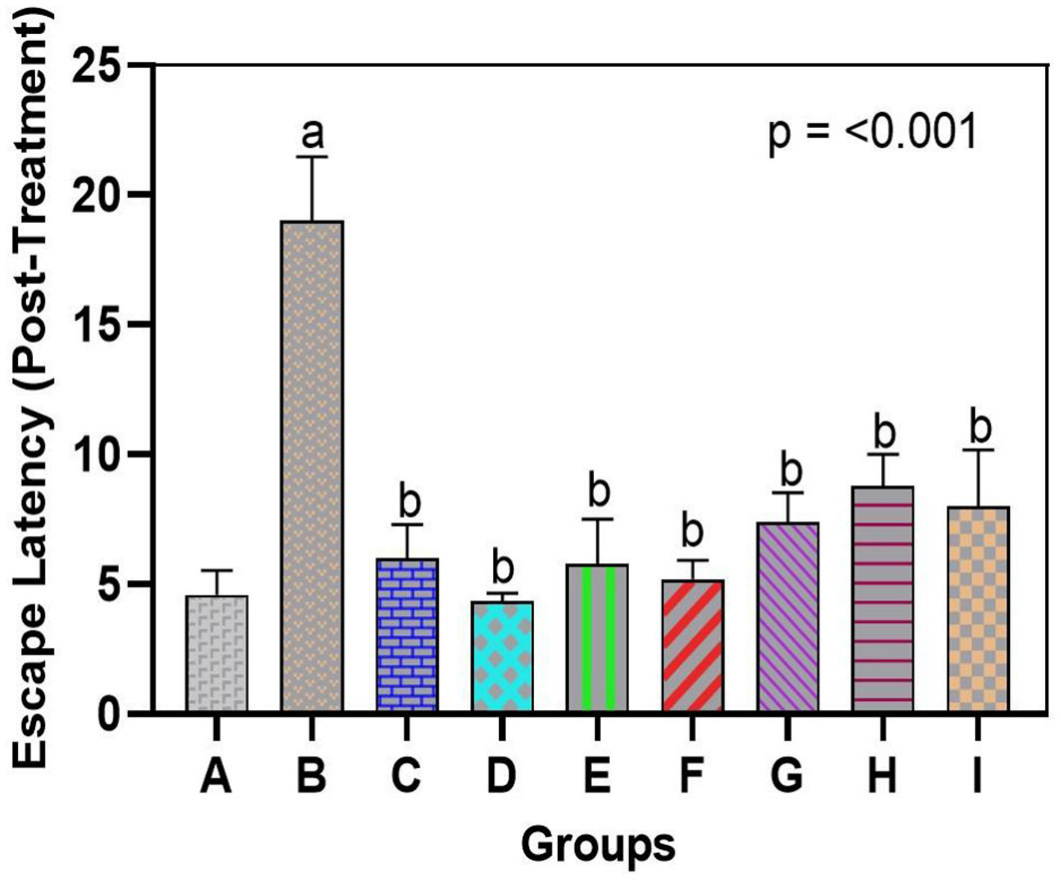
Bromelain improves spatial learning and memory in arsenic-exposed rats. Escape latency (time to find the hidden platform) was significantly longer in arsenic-only rats vs. controls. Bromelain (low, medium, high dose) reduced latency, with high-dose effects comparable to donepezil. Data are mean ± SEM. ^a^p < 0.05 vs. control; ^b^p < 0.05 vs. arsenic-only; ^c^p < 0.05 vs. low-dose bromelain; ^d^p < 0.05 vs. medium-dose bromelain; ^e^p < 0.05 vs. high-dose bromelain; ^f^p < 0.05 vs. donepezil.

There was a significant decline in spatial learning and memory after exposure to arsenic, but the administration of bromelain restored cognitive performance. The morris water maze test in the arsenic-only group (Group B) demonstrated escape latency that was significantly longer than that of normal rats (Group A), signaling major learning and memory impairment in this group. On the last day of training, arsenic-exposed rats spent significantly more time finding the hidden platform (escape latency 55–60 s) than control rats (20–25 s). These arsenic-only rats generally took a considerably longer time to discover the former position of the platform than they had done earlier or failed within 60 seconds, indicating poor retention of spatial memory. In contrast, rats administered bromelain (Groups C, D, and E) escaped in a similar fashion to the controls; escape latencies were as short and did not vary significantly over the period of training as those of Group A, demonstrating that bromelain alone did not have a negative impact on learning or memory. Interestingly, bromelain therapy following exposure to arsenic (Groups F-H) remarkably enhanced maze retention scores compared with untreated arsenic rats. At the end of treatment, the arsenic + bromelain group exhibited decreased escape latencies (p < 0.05, compared with Group B) and effectively countered the arsenic-induced deficit ( Figure 1). Medium-dose bromelain (10 mg/kg, Group G) was remarkable, and the mean escape times were nearly equal to those of the controls. The group administered a high dose of bromelain (15 mg/kg, Group H) showed a shorter escape latency than the donepezil group (Group I) during memory retrieval. Quantitatively, the mean time taken to reach the target was approximately 15 per cent less in the case of high-dose bromelain rats compared to donepezil-treated rats, but this was not significant. Unsurprisingly, donepezil treatment also significantly improved performance compared to arsenic only (p < 0.01), although bromelain at high doses had an even more significant effect on spatial memory. This finding indicates that bromelain has the potential to salvage cognitive dysfunction caused by arsenic and that it is as effective as the conventional medication donepezil. Notably, there were no rats on bromelain-only who demonstrated prolonged latencies or abnormal behavior, indicating that there was no evident adverse effect on cognition with bromelain at the doses used, at least not at inference levels.

### NMDA receptor NR2A and glutamate transporter expression

**Figure 2.**
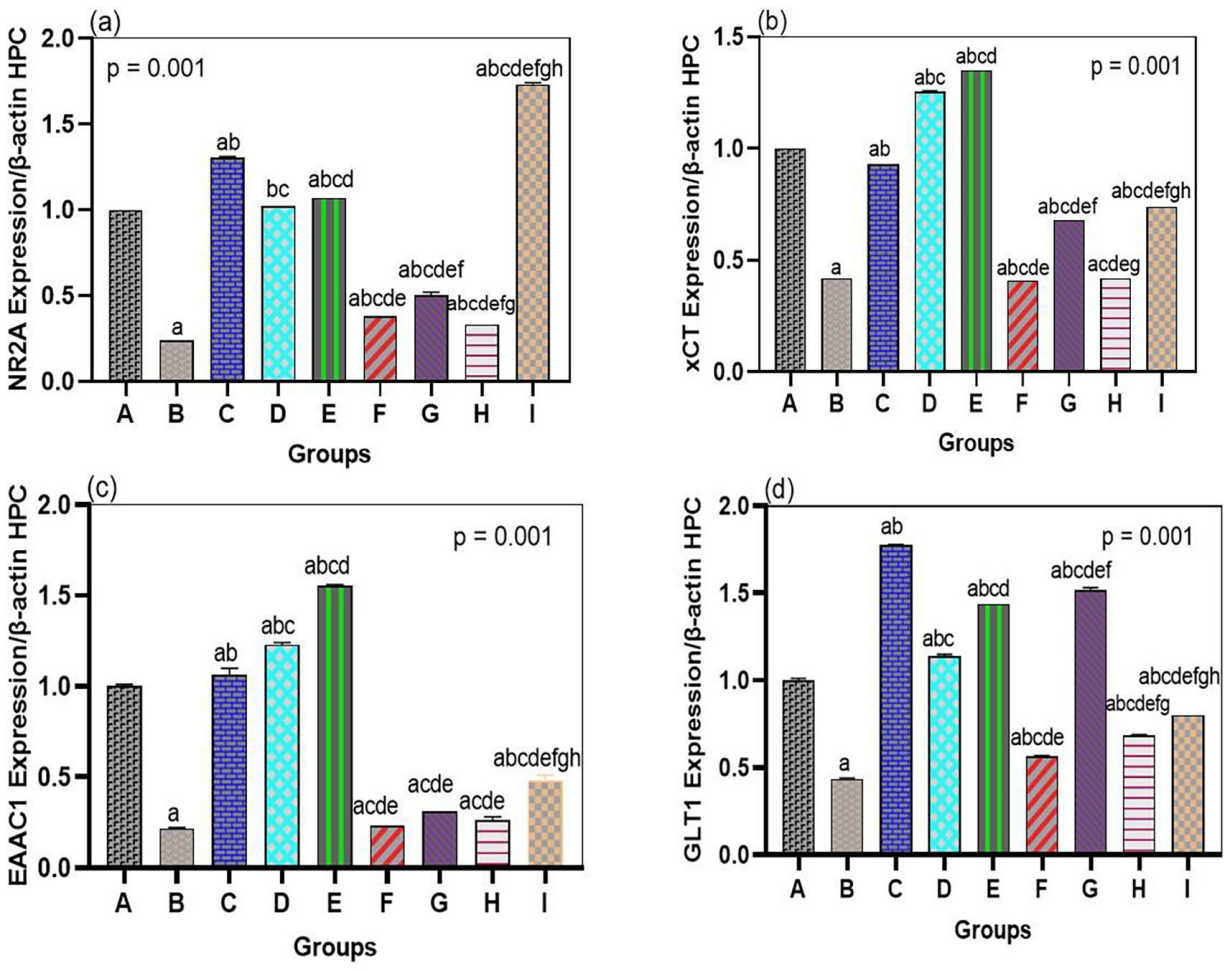
Bromelain upregulates NMDA receptor subunit NR2A and glutamate transporter genes in the hippocampus. mRNA levels of NR2A, xCT, EAAC1, and GLT1 were downregulated by arsenic vs. control. Bromelain (low, medium, high dose) significantly restored expression, with the medium dose showing the strongest effect, approaching control values. Data are mean ± SEM. ^a^p < 0.05 vs. control; ^b^p < 0.05 vs. arsenic-only; ^c^p < 0.05 vs. low-dose bromelain; ^d^p < 0.05 vs. medium-dose bromelain; ^e^p < 0.05 vs. high-dose bromelain; ^f^p < 0.05 vs. donepezil.

**Figure 3.**
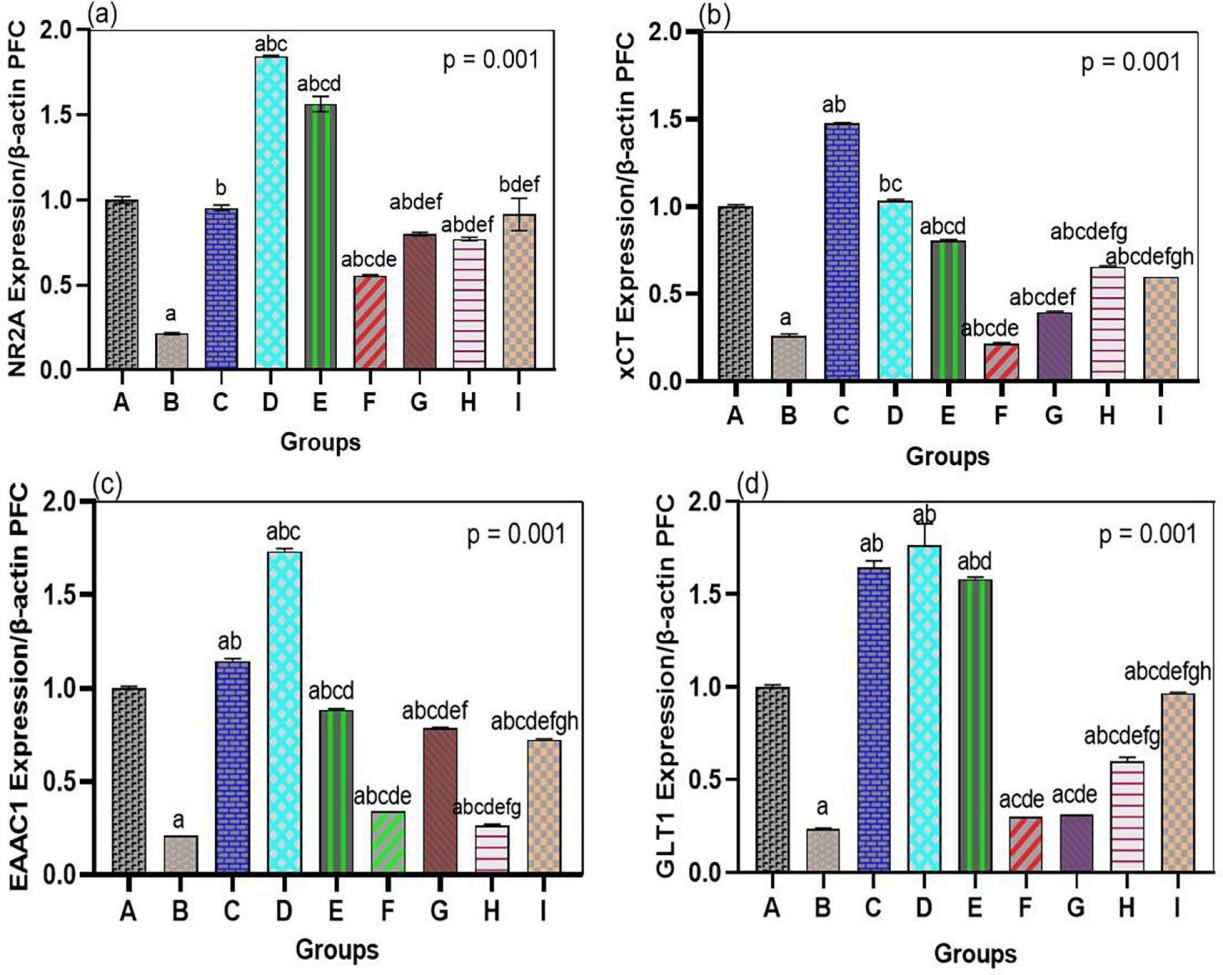
Bromelain upregulates NR2A and transporter genes in the prefrontal cortex. Arsenic suppressed mRNA expression of NR2A, xCT, EAAC1, and GLT1, while bromelain (notably high dose) significantly enhanced expression. Effects were dose-dependent, with strong upregulation of xCT and GLT1. Donepezil generally produced higher expression, though bromelain showed stronger effects on EAAC1 and xCT. Data are mean ± SEM. ^a^p < 0.05 vs. control; ^b^p < 0.05 vs. arsenic-only; ^c^p < 0.05 vs. low-dose bromelain; ^d^p < 0.05 vs. medium-dose bromelain; ^e^p < 0.05 vs. high-dose bromelain; ^f^p < 0.05 vs. donepezil.

The neurotoxic effects of arsenic were linked to the general downregulation of glutamate transporters and receptor genes in both the hippocampus and prefrontal cortex, which bromelain treatment notably overcame. In our arsenic-only rats (Group B), mRNA levels of the NMDA receptor NR2A subunit, the transporters GLT1, EAAC1, and xCT were significantly reduced in both brain regions compared to those in the normal controls (see Figures 2 and 3). This validates that the transcriptional regulation of major glutamate-handling proteins is affected by arsenic exposure, as previously reported. In quantitative terms, arsenic hippocampal NR2A expression was reduced by ~40% of control, GLT1 by ~50%, EAAC1 by ~45%, and xCT by ~60% of control (all p < 0.01 compared with Group A). The same was true for the prefrontal cortex. These changes support the concept that arsenic aggravates glutamate uptake and receptor operations, resulting in a condition that is susceptible to excitotoxicity.

When bromelain was used alone (Groups C, D, and E), minor upregulation of genes related to glutamate was observed relative to the control experiments. When arsenic was absent, low-dose bromelain tended to elevate NR2A, GLT1, EAAC1, and xCT expression by ~10-20% compared to controls, whereas medium- and high-dose bromelain led to minor but statistically significant alterations in the expression of some genes (p < 0.05, vs. Group A subset of targets). Although these modifications in br-only rats were small, they indicate that bromelain has regulatory effects on the expression of neurotransmitter genes, even under normal conditions. Remarkably, this level of upregulation did not result in any cognitive changes, as the bromelain-only rats exhibited normal behavior in the MWM, suggesting that the gene modulatory effects of bromelain are positive but not deleterious.

Most importantly, mRNA levels of NR2A, GLT1, EAAC1, and xCT were strongly enhanced by bromelain therapy after arsenic exposure (Groups F, G, and H) compared to those in the arsenic alone group ( Figures 2 and 3). Bromelain at any dose significantly increased the expression of these genes in the hippocampus (p < 0.01 vs. Group B). The medium dose (10 mg/kg) proved especially effective, as most of the mRNA levels were close to or exceeded the control levels. For example, hippocampal NR2A expression in the medium-dose group was ~95% of the control (a 2.3-fold increase compared to the presence of arsenic-only), whereas GLT1 was ~110% of the control (2-fold increase compared to arsenic-only). Small and large doses of bromelain also brought about considerable changes, but the medium dose resulted in the greatest overall performance in the hippocampus. Surprisingly, the dose-response was slightly different in the prefrontal cortex: high-dose bromelain demonstrated the highest upregulation of xCT and GLT1 (even surpassing the same effect produced by the medium dose), but the upregulation of NR2A and EAAC1 did not increase further with the high dose. This indicated a localized, dose-dependent effect of bromelain on gene regulation. While moderate dosing could best target hippocampal neurons (where NR2A plays a significant role in synaptic plasticity), higher doses could also be effective in enhancing cortical astrocytic function (reported by an increase in xCT and GLT1 in the cortex). Regardless of dose, bromelain-treated arsenic groups demonstrated a significant upregulation in all measured genes compared to the corresponding untreated arsenic group, demonstrating a definite reversal of the suppressive effects of arsenic.

For comparison, these glutamate-related genes were also upregulated by donepezil treatment (Group I) administered after arsenic. Among all groups, donepezil showed the greatest expression of NR2A, GLT1, EAAC1, and xCT in both the hippocampus and cortex, and it was stronger than the effect of bromelain in most drug conditions (p < 0.05 among donepezil and bromelain high-dose conditions when tested on NR2A and GLT1). For example, the hippocampal NR2A level in the donepezil group was approximately 1.3-fold higher than that in the medium bromelain group. This was not surprising since the positive effect of donepezil on glutamatergic neurotransmission was achieved indirectly by increasing cholinergic neurotransmission and neurotrophic support. Nonetheless, some interesting findings were that bromelain performed better than donepezil in some of the measures. In the prefrontal cortex, the high-dose bromelain group showed greater EAAC1 and xCT than donepezil. Cortical xCT mRNA in Group H was approximately 20 percent greater than Group I, and EAAC1 mRNA was approximately 15 percent greater, indicating that bromelain might have an exclusive benefit in regulating glial antioxidant transport systems (xCT) and cortical neuronal glutamate uptake. These minuscule disparities allude to a possibly different condition of bromelain that may supplement or surpass the outcomes of typical treatment in particular brain areas. Collectively, the gene expression data show that bromelain significantly prevents arsenic-induced repression of essential excitatory signaling components, and can therefore restore the potential of glutamate clearance and the normal functioning of NMDARs.

### Glutamate levels in brain tissue

In line with the results of gene and behavioral analyses, the use of bromelain as a treatment reversed the arsenic-induced imbalance in glutamate homeostasis. Exposure to arsenic results in a high accumulation of glutamate in the brain tissue. In the hippocampal homogenates (and cortical homogenates), the arsenic-only group (Group B) had an increased concentration of glutamate compared to the control group ( Figures 2 and 3). Arsenic-only rats exhibited ~1.8-fold greater levels of glutamate than controls on average (p < 0.01), indicative of impaired clearance and excitotoxic saturation. This is consistent with the idea that arsenic produces glutamate dyshomeostasis, likely as a result of impaired transporter activity and hyperrelease. The glutamate ELISA, In feasible terms, unraveled concentrations of approximately 12-15 micromole/gram in arsenic brains in comparison to ~7-8 micromole/gram controls.

Glutamate levels were not significantly different between the bromelain-only groups (C, D, E) and the control group. Indeed, glutamate levels in these rats were low (similar to Group A), which means that bromelain alone does not exhaust glutamate below normal levels or leads to its abnormal accumulation. This is a vital safety assurance that bromelain has no effect on destabilizing baseline neurotransmitters.

**Figure 4.**
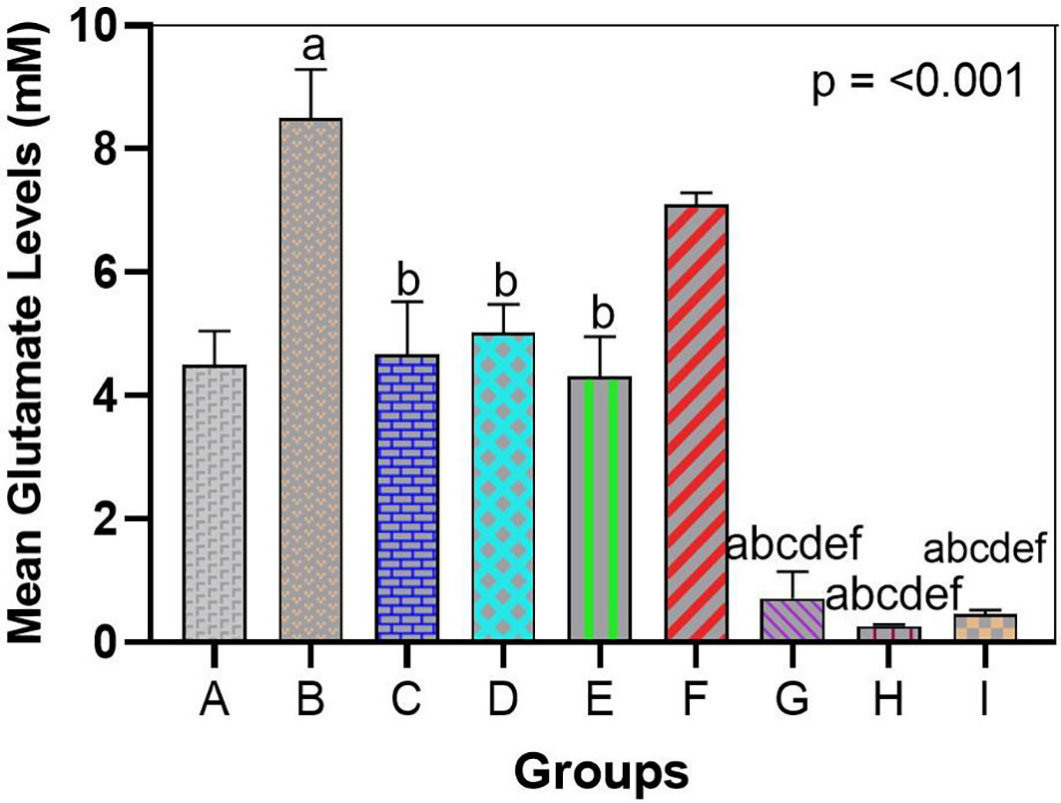
Bromelain reduces arsenic-elevated glutamate in hippocampal tissue. Arsenic significantly increased glutamate vs. control, indicating excitotoxicity. Bromelain lowered glutamate dose-dependently, with high dose normalizing levels and outperforming donepezil. Data are mean ± SEM. ^a^p < 0.05 vs. control; ^b^p < 0.05 vs. arsenic-only; ^c^p < 0.05 vs. low-dose bromelain; ^d^p < 0.05 vs. medium-dose bromelain; ^e^p < 0.05 vs. high-dose bromelain; ^f^p < 0.05 vs. donepezil.

Glutamate concentration was also significantly decreased in arsenic-treated rats administered bromelain (Groups F and G) compared to arsenic only in Group D. A trend of reduced glutamate with low-dose bromelain was observed ( Figure 4); however, the difference was not significant (glutamate = 12 (micro sign) m/g in Group B vs. 15 (micro sign) m/g in Group A, p = 0.09). The middle dose produced a strong adjustment (p < 0.05, compared with Group B) and re-approached the norm of glutamate concentration (~9-10 mU/g). The strongest effect was achieved with high-dose bromelain, whose Glu content was close to that of the control (~8 μmol/g, not significantly different from the control) and significantly lower than that in the medium-dose and arsenic-only groups. Indeed, high-dose bromelain decreased brain glutamate levels even more than donepezil (Group I glutamate ~9 μmol/g compared with Group H ~8 μmol/g). Regardless of the fact that both treatments reduced the influence of glutamate accumulation, the large amount of bromelain was more effective in relation to donepezil. This indicates the effectiveness of bromelain in increasing glutamate clearance or decreasing the release of glutamate in toxic states. Statistical comparisons revealed that the glutamate level in Group H was significantly lower than that in Group I (p < 0.05), proving that bromelain has a strong effect when administered at high doses.

Taken together, bromelain can normalize the neurochemical imbalance caused by arsenic. Bromelain probably prevents neuronal over-excitation by preventing glutamate accumulation with excitotoxic effects. The glutamate results are consistent with the increases in transporter expression; when bromelain increased the transporter expression of GLT1, EAC, and xCT, the ability of the brain to remove or recycle glutamate was enhanced, causing reduced steady-state levels of glutamate tissue. This biochemical finding supports the behavioral findings, suggesting that there is a mechanistic relationship between the improved cognitive agent bromelain and restored glutamate homoeostasis.

## Discussion

Research has shown that bromelain, a natural substance, has a strong defense effect against arsenic-induced cognitive dysfunction by regulating glutamate neurotransmission. Exposure to arsenic results in major impairments of spatial learning and memory with associated glutamate excitotoxicity and suppression of key glutamate transporters and glutamate NMDAR subunits. These findings are congruent with an understanding of the pathophysiology of arsenic neurotoxicity. ^47, 48^ Excessive extracellular glutamate and overactivation of NMDARs mediated by arsenic lead to dysfunction of synapses and neuronal damage, which presents as cognitive impairments. ^40, 44^ We found that rats exposed to arsenic only exhibited long escape latencies in the MWM and were barely able to remember the position of the platform indicating extreme inhibition of hippocampus-mediated memory. Simultaneously, they had increased glutamate levels in their brain tissues (which is an indicator of poor glutamate clearance) and the expression of NR2A, GLT1, EAAC1, and xCT significantly declined. Interestingly, NR2A subunits are downregulated: NR2A-containing NMDA receptors play an essential role in synaptic plasticity and memory consolidation, and their deficiency is probably one of the causes of cognitive dysfunction. In addition, the decreased levels of GLT1 and some other transporters support previous research in which arsenic was shown to downregulate glutamate transportation, thereby increasing the risk of excitotoxicity. ^42, 43^ Similar conclusions were drawn by Hubbard and Binder, ^49^ who revealed that exposure to arsenic inhibits the expression of GLT1/EAAT2 and xCT, and as a result, suppresses glutamate uptake. These findings further support learning and memory impairment as an attribute of arsenic neurotoxicity, which is primarily due to glutamate dysregulation.

Most importantly, bromelain therapy successfully reversed these arsenic-mediated alterations. It was found that there was a significant improvement in the performance levels of the rats treated with bromelain in the Morris water maze, which indicates that they had recovered their ability to perform cognitive tasks. The bromelain-dose-dependent contribution to the reduction of escape latencies was spectacular: all doses of bromelain-treated groups showed better results in comparison to arsenic-only treatment, and even the highest dose of bromelain groups showed shorter escape latencies than the dose-of-donepezil-treated group. This indicates that the cognitive enhancing effect of bromelain can either be comparable or even surpass the effect of a normal cognitive-enhancing drug such as donepezil in arsenic-induced deficits. In addition, bromelain alone did not affect cognition, which agrees with a recent observation that bromelain can be used to improve memory in healthy rodents without side effects. Kumar et al. ^38^ observed similar results when bromelain supplementation enhanced spatial learning in mice, proving that, according to our observations, bromelain intrinsically possesses pro-cognitive properties.

The most impressive result was the normalization of brain glutamate levels conferred by bromelain. Bromelain significantly decreased the increased glutamate level, which was induced by arsenic to almost the control level, particularly at 15 mg/kg. This decrease is probably a direct result of the upregulation of glutamate transporters. In elevating the expression of GLT1 (predominantly astrocytic) and EAAC1 (neuronal), bromelain therefore serves to augment extracellular glutamate clearance and reduce excitotoxic stress. A correlation between transporter action and glutamate concentration has been firmly established; restoring a glutamate transporter partially reduces extracellular glutamate and rescues neurons. ^17, 18, 50, 51^ This is in line with our findings because the upregulation of bromelain transport was associated with reduced glutamate and enhanced cognition. According to Turski and Ikonomidou, ^52^ it is imperative to maintain low extracellular glutamate levels to mediate excitotoxicity, which is a precipitator of neurodegeneration. The glutamate-reducing effect of bromelain is consistent with an earlier study by Bakare and Owoyele, ^53^ who identified similar effects of bromelain in a pain model linked to the attenuation of neuropathic pain signaling. By decreasing glutamate levels in our study, bromelain most likely stopped the vicious cycle of NMDAR overactivation and neuron loss that arsenic would have reinforced. This mechanistic knowledge of how bromelain increases glutamate clearance provides novel information on the neuroprotective effects of bromelain.

Another important finding was the upregulation of the NMDAR NR2A subunit by bromelain. The expression of the NR2A subunit was severely downregulated in arsenic-only rats, which is concerning because loss of NR2A affects the process of synaptic plasticity and may alter NMDAR signaling to become pro-death (a high ratio of NR2B to NR2A enhances excitotoxicity). The ability of bromelain, especially medium doses, to establish normal NR2A levels in the hippocampus and cortex is associated with better memory. This implies that bromelain can protect or restore the normal NMDAR composition and performance after toxic stress. Surprisingly, an intermediate dose of bromelain was the best dose for NR2A, suggesting a hormetic or optimal dose response. Perhaps a moderate level of bromelain is the optimal amount to engage in the intracellular signaling (CREB or BDNF) pathway to facilitate NR2A expression, and excessive amounts do not offer any extra benefit in that respect. Ladagu et al. ^54^ noted that neurotoxicity caused by metals can be addressed by specific interventions through the regulation of NMDAR subunits. The implication of our data is that bromelain can restore the balance of NMDAR subunits closer to physiological values, thus contributing to synaptic resilience. The bromelain-mediated enhancement of NR2A expression correlates well with enhanced cognitive performance; NR2A-bearing NMDARs play a significant role in long-term potentiation and memory consolidation, and their restoration probably promoted the relearning that was seen in bromelain-treated rats.

The region-specific differences evident (medium vs. high dose effects in the hippocampus vs. cortex) are also important. A region of the brain that is important in spatial memory, namely the hippocampus, was most effectively stimulated by 10 mg/kg bromelain in both gene expression and memory, whereas the prefrontal cortex (related to executive function and working memory) showed the highest transporter upregulation at 15 mg/kg. This could be related to cell population differences: the cortex is thickly covered in an astrocytic network that could take a greater dose of bromelain to drive up xCT and (GLT1) expression, whereas the hippocampus (perhaps with a different blood-brain barrier permeability or sensitivity of enzymes) demonstrated an effect saturation at the intermediate dose. It may also be that ridiculously large bromelains may tap into feedback mechanisms that at least tone down some gene expression in the hippocampus. Nevertheless, it is also clear that both areas enjoyed the positive effects of bromelain, highlighting its central action.

From a therapeutic perspective, our results show that bromelain is a promising agent for the attenuation of excitotoxic brain injury. The multimodal actions of bromelain (antiinflammatory, antioxidant (implied by others), ^55– 57^ and are now shown to include glutamatergic regulation) make bromelain a promising neuroprotective agent. In contrast to drugs that affect a single pathway, bromelain would provide an overall protective effect: it decreased oxidative stress markers in other trials and corrected neurotransmitter balance in our trial. Moreover, bromelain is a low-risk oral nutraceutical that may be beneficial for any chronic treatment or at-risk populations. The capability of this model to compete with that of donepezil is outstanding. Donepezil acts primarily to enhance cholinergic signaling; however, its effectiveness in this regard is an important indicator of the role of glutamatergic pathways in mental health and evidence that bromelain exerts its cognitive effects via an alternate mechanism that is potentially complementary.

It is possible to consider the mechanisms through which bromelain can produce these transcriptional effects. Bromelain can modify some of the essential signaling pathways that determine the existence of glutamate transporters. That is, inhibition of inflammatory cytokines via the NF-kappaB pathway, or upregulation of GLT1 by nuclear receptor activation, such as the peroxisome proliferator activated receptor. Bromelain could also induce anti-inflammatory effects by lowering TNF- a, IL-1 beta and other cytokines, which can help to alleviate inflammation-induced inhibition of transporter gene expression. Bromelain has also been noted to stimulate extracellular signal receptor kinase (ERK) pathways and elevate the levels of brain-derived neurotrophic factor (BDNF) in stressed animals; hence, a higher expression of synaptic proteins might follow. Moreover, the antioxidant effect of bromelain may stimulate the Nrf2 pathway, which is associated with an elevated expression of xCT (because xCT is a constituent of the glutathione production complex). In fact, Eraky et al. (2023) ^37^ demonstrated that bromelain altered the TXNIP/Nrf2 axis in an Alzheimer model. Should bromelain stimulate Nrf2, which would result in the observed increase in xCT expression because Nrf2 induces the transcription of antioxidant response elements in genes such as SLC7A11 (xCT). These hypotheses may require additional molecular experimentation, although it is clear that bromelain achieves an overall neuroprotective effect by acting on many cell signaling pathways.

Nevertheless, there are a few limitations to admission despite agreeable outcomes. The sample sizes we considered were relatively small (n = 5 per group), but they provided adequate power to identify differences (increased group sizes would have better statistics and would have enabled stratification by sex (we had used males only in the current study). Second, the protocol of our study was performed at the mRNA level of gene expression; it is compelling to verify whether there are corresponding elevations in the protein levels of NR2A and transporter proteins (e.g., via western blot or immunohistochemistry). Third, the model of arsenic exposure, although applicable, is an acute/subacute toxicity situation. As accumulates its effect in real-life chronic exposure over prolonged periods. It would be interesting to determine whether bromelain has a similar beneficial effect in chronic low-dose exposure to arsenic or other excitotoxic states (such as stroke or epilepsy). Finally, although we have demonstrated that there are associations between the biochemical effects and functional improvements of bromelain, the links lack causality (i.e., we did not investigate the presence of a relationship to the point where reducing glutamate led to cognitive improvement). In future studies, the causal effect of specific transporters or receptors on the actions of bromelain could be proven by employing pharmacological blocking or gene knockdown.

Taken together, our results show that bromelain may be a complex neuroprotective agent. It not only enhances behavioral performance in an arsenic-induced cognitive impairment model but also ameliorates the underlying glutamatergic imbalance by increasing transporters and stabilizing NMDAR subunits. This example of an integrative mechanism of action also sets bromelain apart from the more conventional forms of treatment that may coincide with influencing only a particular factor (donepezil exerts its effects on the levels of acetylcholine but does not directly affect the glutamate systems). The fact that administration of bromelain (or bromelain-containing supplements) has the ability to improve spatial memory in rats and remove risk factors of excitotoxicity indicates that this compound may be investigated as an adjunctive therapy in diseases characterized by glutamate excitotoxicity (such as neurodegenerative diseases), as well as in populations at risk of environmental toxin exposure (possibly as a preventive factor).

## Conclusion

In conclusion, this study demonstrates that bromelain has neuroprotective and cognitive-enhancing activity in a rat model of arsenic-mediated neurotoxicity. Treatment with bromelain enhanced spatial learning and memory abilities and was associated with the regulation of excitatory neurotransmission, which increased the NMDA receptor NR2A subunit and glutamate transporters (GLT1, EAAC1, and xCT) in the hippocampus and prefrontal cortex, and thus, may induce elimination of glutamate and avoid excitation toxicity. Such molecular adaptations assist in the normalization of brain glutamate levels and maintenance of synaptic integrity in response to toxic stress. The effectiveness of bromelain was dependent on the dose, giving it equivalent or similar abilities at higher doses than donepezil in selected measures and validating its use as a treatment option. Since bromelain is naturally based and has a good safety history, our results provide the possibility of its application in the reversal of cognitive impairments induced by environmental toxins or in neurodegenerative disorders, such as glutamate maladaptation. Future studies are needed to identify the signaling pathways through which bromelain alters gene expression, as well as its long-term potential and utility in other models of cognitive dysfunction. Bromelain appears to be a potentially new way to protect cognitive health, which acts through different mechanisms of neurotoxicity, as seen in the case of arsenic exposure.

### Declarations

**Clinical trial number**: Not applicable.

### Ethics approval and consent to participate

The experimental protocol was approved by the College of Medicine, University of Nigeria Institutional Animal Care and Use Committee (IACUC) (ethics approval no. COMHREC/2025/01/025). All procedures were conducted in compliance with the ethical standards.

## Data Availability

Data generated can be accessed via: DOI: https://doi.org/10.6084/m9.figshare.29903342.v2. ^58^ Data are available under the terms of the Creative Commons Attribution 4.0 International License (CC BY 4.0).

## References

[1] Sceniak MP : An autism-associated mutation in GluN2B prevents NMDA receptor trafficking and interferes with dendrite growth. *J. Cell Sci.* 2019;132(20). 10.1242/JCS.232892 31548203

[2] *NMDA Receptors in Health and Disease.* IntechOpen; Accessed: Jun. 27, 2025. Reference Source

[3] Huang Y-Q Huang Y-Q : NMDA Receptors in Health and Disease. Apr. 2024. 10.5772/INTECHOPEN.114003

[4] Liu J Chang L Song Y : The role of NMDA receptors in Alzheimer’s disease. *Front. Neurosci.* Feb. 2019;13(FEB):425433. 10.3389/FNINS.2019.00043/XML/NLM PMC6375899 30800052

[5] Taghibiglou C Khalaj S : Cholesterol and Fat Metabolism in Alzheimer’s Disease. *Drug Discov. Approaches Treat. Neurodegener. Disord. Alzheimer’s Dis.* 2017;161–193. 10.1016/B978-0-12-802810-0.00009-X

[6] Zhang X Cai W Wang C : N-methyl-D-aspartate receptors (NMDARs): a glutamate-activated cation channel with biased signaling and therapeutic potential in brain disorders. *Pharmacol. Ther.* Sep. 2025;273:108888. 10.1016/J.PHARMTHERA.2025.108888 40412765

[7] Jewett BE Thapa B : Physiology, NMDA Receptor. *StatPearls.* Dec. 2022. Accessed: Jun. 27, 2025. Reference Source 30137779

[8] Dong B Yue Y Dong H : N-methyl-D-aspartate receptor hypofunction as a potential contributor to the progression and manifestation of many neurological disorders. *Front. Mol. Neurosci.* 2023;16:1174738. 10.3389/FNMOL.2023.1174738 37396784 PMC10308130

[9] Guan S : Deciphering the dual role of N-methyl-D-Aspartate receptor in postoperative cognitive dysfunction: A comprehensive review. *Eur. J. Pharmacol.* May 2024;971:176520. 10.1016/J.EJPHAR.2024.176520 38527701

[10] O’Donovan SM Sullivan CR McCullumsmith RE : The role of glutamate transporters in the pathophysiology of neuropsychiatric disorders. *npj Schizophr.* Sep. 2017;3(1):14–32. 10.1038/s41537-017-0037-1 28935880 PMC5608761

[11] Martinez-Lozada Z Ortega A : Milestone Review: Excitatory amino acid transporters – Beyond their expected function. *J. Neurochem.* May 2023;165(4):457–466. 10.1111/JNC.15809 36920226

[12] Litwack G : Membrane Transport. *Hum. Biochem.* 2018;553–589. 10.1016/B978-0-12-383864-3.00018-1

[13] Malik AR Willnow TE : Excitatory Amino Acid Transporters in Physiology and Disorders of the Central Nervous System. *Int. J. Mol. Sci.* Nov. 2019;20(22):5671. 10.3390/IJMS20225671 31726793 PMC6888459

[14] Pajarillo E Rizor A Lee J : The role of astrocytic glutamate transporters GLT-1 and GLAST in neurological disorders: potential targets for neurotherapeutics. *Neuropharmacology.* Dec. 2019;161:107559. 10.1016/J.NEUROPHARM.2019.03.002 30851309 PMC6731169

[15] Martis RM Knight LJ Donaldson PJ : Identification, Expression, and Roles of the Cystine/Glutamate Antiporter in Ocular Tissues. *Oxidative Med. Cell. Longev.* Jan. 2020;2020(1):1–10. 10.1155/2020/4594606 32655769 PMC7320271

[16] Ziar R Tesar PJ Clayton BLL : Astrocyte and oligodendrocyte pathology in Alzheimer’s disease. *Neurotherapeutics.* Apr. 2025;22(3):e00540. 10.1016/J.NEUROT.2025.E00540 39939240 PMC12047399

[17] Castillo-Vazquez SK : Glutamatergic Neurotransmission in Aging and Neurodegenerative Diseases: A Potential Target to Improve Cognitive Impairment in Aging. *Arch. Med. Res.* Sep. 2024;55(6):103039. 10.1016/J.ARCMED.2024.103039 38981341

[18] Rimmele TS : Neuronal Loss of the Glutamate Transporter GLT-1 Promotes Excitotoxic Injury in the Hippocampus. *Front. Cell. Neurosci.* Dec. 2021;15:788262. 10.3389/FNCEL.2021.788262/XML/NLM 35035352 PMC8752461

[19] Ramandi D Salmani ME Moghimi A : Pharmacological upregulation of GLT-1 alleviates the cognitive impairments in the animal model of temporal lobe epilepsy. *PLoS One.* Jan. 2021;16(1):e0246068. 10.1371/JOURNAL.PONE.0246068 33507976 PMC7842975

[20] Fontana ACK : Current approaches to enhance glutamate transporter function and expression. *J. Neurochem.* Sep. 2015;134(6):982–1007. 10.1111/JNC.13200;PAGE:STRING:ARTICLE/CHAPTER 26096891

[21] Anyanwu E : Fisetin attenuates AlCl3-induced neurodegeneration by modulating oxidative stress and inflammatory cytokine release in adult albino wistar rats. *Toxicol. Rep.* Dec. 2024;13:101812. 10.1016/J.TOXREP.2024.101812 39624221 PMC11609245

[22] Obasi KK Anyanwu GE : Fisetin potentiates anxiolysis in rat models of aluminium chloride-induced Alzheimer-like disease. *J. Krishna Inst. Med. Sci. Univ.* 2022.

[23] Oviosun A Oviosun EC Nto NJ : Zingerone Attenuates Cadmium-Induced Neuroinflammation, Oxidative Stress and Cognitive Deficit on the Prefrontal Cortex of Adult Wistar Rats. *J. Exp. Pharmacol.* 2025;17:323–341. 10.2147/JEP.S519571 40535155 PMC12174926

[24] Oviosun A Godson Anyanwu E Chidinma Oviosun E : Ameliorative effect of zingerone on cadmium-induced nephrotoxicity in adult wistar rats. *Int. J. Plant Based Pharm.* Nov. 2024;4(2):118–124. 10.62313/ijpbp.2024.238

[25] Oviosun A Anyanwu EG Oviosun EC : Title: Histochemical, immunohistochemical and behavioural demonstration of the potential of Zingerone to ameliorate cognitive decline in animal model. *Alzheimers Dement.* Dec. 2023; vol.19(S13). 10.1002/ALZ.075925

[26] Emeka AG Augustine O Chidinma OE : Zingerone improves memory impairment in Wistar rats exposed to cadmium via modulation of redox imbalance. Accessed: Jun. 28, 2025. Reference Source

[27] Anyanwu GE Umeano AV Ojiakor VO : Morin Mitigates Methamphetamine-Induced Neurotoxicity: Effects on Motor and Cognitive Function. *J. Exp. Pharmacol.* 2025;17:307–321. 10.2147/JEP.S498984 40524867 PMC12168913

[28] Egwuatu IA : Ameliorative Effects of 5-7, Dihydroxy Flavone (Chrysin) on Hippocampus of Wistar Rats with Doxorubicin-induced Cognitive Impairment. *J. Complement. Altern. Med. Res.* Jun. 2023;22(2):49–61. 10.9734/JOCAMR/2023/V22I2456

[29] Oria RS : Modulatory Role of Curcumin on Cobalt-Induced Memory Deficit, Hippocampal Oxidative Damage, Astrocytosis, and Nrf2 Expression. *Neurotox. Res.* 2023;41:201–211. 10.1007/s12640-023-00635-6 36692684

[30] Oria RS Anyanwu GE Nto JN : Curcumin abrogates cobalt-induced neuroinflammation by suppressing proinflammatory cytokines release, inhibiting microgliosis and modulation of ERK/MAPK signaling pathway. *J. Chem. Neuroanat.* 2024;137:102402. 10.1016/j.jchemneu.2024.102402 38428651

[31] Varilla C Marcone M Paiva L : Bromelain, a Group of Pineapple Proteolytic Complex Enzymes (Ananas comosus) and Their Possible Therapeutic and Clinical Effects. A Summary. *Foods.* Oct. 2021;10(10):2249. 10.3390/FOODS10102249 34681298 PMC8534447

[32] Verma V Singhal G Joshi S : Plant extracts as enzymes. *Plant Extr. Appl. Food Ind.* Jan. 2021;209–223. 10.1016/B978-0-12-822475-5.00009-0

[33] Vasiljevic T : Pineapple. *Valorization Fruit Process. By-products.* Jan. 2020;203–225. 10.1016/B978-0-12-817106-6.00010-1

[34] Parasuraman R Jayamurali D Manoharan N : Neuroprotective effect of bromelain on BDNF-TRKB signalling pathway in chronic unpredictable stress-induced depression model. *Beni-Suef Univ. J. Basic Appl. Sci.* Dec. 2024;13(1):1–19. 10.1186/S43088-024-00482-0/FIGURES/11

[35] Rostamian S Raeisi E Heidari-Soureshjani S : Neuroprotective Effects of Bromelain on the Common Neurodegenerative Diseases: A Systematic Review. *Neurochem. J.* Jan. 2024;17(4):715–726. 10.1134/S1819712423040256

[36] Hsu JCN : Bromelain prevents Alzheimer’s disease progression by suppressing oxidative stress and upregulating apolipoprotein A1 in 5x familial Alzheimer’s disease transgenic mice. *J. Agric. Food Res.* Jun. 2025;21:101927. 10.1016/J.JAFR.2025.101927

[37] Eraky SM Ramadan NM Abo El-Magd NF : Ameliorative effects of bromelain on aluminum-induced Alzheimer’s disease in rats through modulation of TXNIP pathway. *Int. J. Biol. Macromol.* Feb. 2023;227:1119–1131. 10.1016/J.IJBIOMAC.2022.11.291 36462588

[38] Kumar R : Pharmacological evaluation of bromelain in mouse model of Alzheimer’s disease. *Neurotoxicology.* May 2022;90:19–34. 10.1016/J.NEURO.2022.02.009 35219781

[39] Tyler CR Allan AM : The Effects of Arsenic Exposure on Neurological and Cognitive Dysfunction in Human and Rodent Studies: A Review. *Curr. Environ. Heal. Reports.* Jun. 2014;1(2):132–147. 10.1007/S40572-014-0012-1 24860722 PMC4026128

[40] Karim Y : Dose-dependent relationships between chronic arsenic exposure and cognitive impairment and serum brain-derived neurotrophic factor. *Environ. Int.* Oct. 2019;131:105029. 10.1016/J.ENVINT.2019.105029 31352261

[41] Vázquez Cervantes GI : Mechanisms Associated with Cognitive and Behavioral Impairment Induced by Arsenic Exposure. *Cells.* Nov. 2023;12(21):2537. 10.3390/CELLS12212537 37947615 PMC10649068

[42] Ramos-Chávez LA Rendón-López CRR Zepeda A : Neurological effects of inorganic arsenic exposure: altered cysteine/glutamate transport, NMDA expression and spatial memory impairment. *Front. Cell. Neurosci.* Feb. 2015;9(FEB):21. 10.3389/FNCEL.2015.00021 25709567 PMC4321597

[43] Castro-Coronel Y Del Razo LM Huerta M : Arsenite exposure downregulates EAAT1/GLAST transporter expression in glial cells. *Toxicol. Sci.* Aug. 2011;122(2):539–550. 10.1093/TOXSCI/KFR126 21602192

[44] Luo JH Qiu ZQ Zhang L : Arsenite exposure altered the expression of NMDA receptor and postsynaptic signaling proteins in rat hippocampus. *Toxicol. Lett.* May 2012;211(1):39–44. 10.1016/J.TOXLET.2012.02.021 22421273

[45] Forero MG Hernández NC Morera CM : A new automatic method for tracking rats in the Morris water maze. *Heliyon.* 2023 Jul 15;9(7):e18367. 10.1016/j.heliyon.2023.e18367 37519749 PMC10372735

[46] Sabine S Leonidas F Priyanka R-R : Dissection of rodent brain regions: Guided free-hand slicing and dissection of frozen tissue. Li KW , editor. *Neuroproteomics.* 2nd ed.Vol. 146.2019; pp.7–19. Accessed: Jun. 29, 2025. 10.1007/978-1-4939-9662-9_2 Reference Source

[47] Thakur M Rachamalla M Niyogi S : Molecular mechanism of arsenic-induced neurotoxicity including neuronal dysfunctions. *Int. J. Mol. Sci.* Sep. 2021;22(18). 10.3390/IJMS221810077 34576240 PMC8471829

[48] Medda N Patra R Ghosh TK : Neurotoxic Mechanism of Arsenic: Synergistic Effect of Mitochondrial Instability, Oxidative Stress, and Hormonal-Neurotransmitter Impairment. *Biol. Trace Elem. Res.* Nov. 2020;198(1):8–15. 10.1007/S12011-020-02044-8 31939057

[49] Hubbard JA Binder DK : Targeting glutamate transporter-1 in neurological diseases. *Oncotarget.* 2017;8(14):22311–22312. 10.18632/ONCOTARGET.16374 28423610 PMC5410224

[50] Dahlmanns M Dahlmanns JK Savaskan N : Glial Glutamate Transporter-Mediated Plasticity: System xc -/xCT/SLC7A11 and EAAT1/2 in Brain Diseases. *Front. Biosci. - Landmark.* Mar. 2023;28(3). 10.31083/J.FBL2803057 37005761

[51] Olajide OJ : Hippocampal Degeneration and Behavioral Impairment During Alzheimer-Like Pathogenesis Involves Glutamate Excitotoxicity. *J. Mol. Neurosci.* Jun. 2021;71(6):1205–1220. 10.1007/S12031-020-01747-W 33420680

[52] Turski GN Ikonomidou C : Glutamate as a Neurotoxin. *Handb. Neurotox.* 2022;769–801. 10.1007/978-3-031-15080-7_84

[53] Bakare AO Owoyele BV : Bromelain reduced pro-inflammatory mediators as a common pathway that mediate antinociceptive and anti-anxiety effects in sciatic nerve ligated Wistar rats. *Sci. Rep.* Dec. 2021;11(1):289. 10.1038/S41598-020-79421-9 33432004 PMC7801445

[54] Ladagu AD : Novel NMDA-receptor antagonists ameliorate vanadium neurotoxicity. *Naunyn Schmiedeberg’s Arch. Pharmacol.* May 2020;393(9):1729–1738. 10.1007/S00210-020-01882-6 32388602

[55] Bottega R Persico I De Seta F : Anti-inflammatory properties of a proprietary bromelain extract (Bromeyal ^TM^) after in vitro simulated gastrointestinal digestion. *Int. J. Immunopathol. Pharmacol.* 2021;35:20587384211034690. 10.1177/20587384211034686 34387509 PMC8366142

[56] Pereira IC Sátiro Vieira EE Oliveira Torres LR de : Bromelain supplementation and inflammatory markers: A systematic review of clinical trials. *Clin. Nutr. ESPEN.* Jun. 2023;55:116–127. 10.1016/J.CLNESP.2023.02.028 37202035

[57] Jebur AB El-Demerdash FM Kang W : Bromelain from Ananas comosus stem attenuates oxidative toxicity and testicular dysfunction caused by aluminum in rats. *J. Trace Elem. Med. Biol.* Dec. 2020;62:126631. 10.1016/J.JTEMB.2020.126631 32763766

[58] Emeka A Kpokuechukwu O Augustin A : figshare - credit for all your research - Bromelain Restores Glutamatergic Homeostasis via Regulation of NR2A, GLT-1, EAAC1, and xCT in Arsenic-Induced Cerebral Cortex and Hippocampal Neurotoxicity - Item Edit. Accessed: Aug. 13, 2025. 10.6084/m9.figshare.29903342.v2 Reference Source PMC12790598 41527666

