# Supplementary material for: Bromelain Restores Glutamatergic Homeostasis via Regulation of NR2A, GLT-1, EAAC1, and xCT in Arsenic-Induced Cerebral Cortex and Hippocampal Neurotoxicity
Source: F1000Res. 2025 Dec 31;14:1053. Originally published 2025 Oct 7. [Version 2] doi: 10.12688/f1000research.169308.2 (PMC12790598; doi:10.12688/f1000research.169308.2)
Supplement: Peer review history

# Reviewer report: Reviewer response for version 2

**DOI:** 10.5256/f1000research.194485.r446843

**Published:** 2026-01-29

**Authors:** Tak Pan Wong

**Affiliations:** 1 McGill University, Montreal, Canada

Godson et al., in this revised version of the manuscript, examined the effects of bromelain, a group of enzymes derived from Ananas comosus, on arsenic‑induced glutamatergic dysfunction and cognitive impairment in rats. Previous studies suggest that the neurotoxic effects of arsenic may be mediated through NMDAR‑related excitotoxicity. The authors therefore hypothesized that bromelain, which has been reported to exert neuroprotective effects, could rescue cognitive deficits by modulating the expression of NMDAR subunits and glutamate transporters.

 In this study, adult male rats were exposed to arsenic, which impaired spatial memory performance in the Morris water maze (MWM). Bromelain treatment attenuated these arsenic‑induced cognitive deficits. RT‑PCR analyses revealed that arsenic significantly reduced the expression of the NR2A subunit of the NMDAR as well as several glutamate transporters (xCT, EAAC1, and GLT‑1), and that these effects were partially reversed by bromelain treatment. In addition, the authors used an ELISA‑based assay to quantify glutamate levels in hippocampal homogenates and found that arsenic increased glutamate levels, which were markedly reduced by bromelain treatment as well as by the positive control, donepezil.

 Taken together, the authors concluded that bromelain provides multi‑target neuroprotection against arsenic‑induced neurotoxicity by restoring glutamatergic homeostasis through upregulation of NR2A and glutamate transporters.

 Exposure to arsenic from contaminated groundwater and food remains a major health issue worldwide, particularly in developing countries. Studies that contribute to a better understanding and treatment of arsenic‑related neurotoxicity are therefore important. The authors have clearly explained the rationale of the study. The experiments are described in detail and include appropriate controls, as well as a positive control group (donepezil).

 My major concerns relate to the choice of methods used to detect glutamate levels and the unclear presentation of most of the data. Glutamate measured from homogenized tissue was used to represent extracellular glutamate levels associated with neurotoxicity. This assumption is unfortunately incorrect. Glutamate is present intracellularly and also functions as an amino acid beyond its role as a neurotransmitter. Measuring glutamate levels in tissue homogenates reflects total tissue glutamate content rather than extracellular glutamate turnover, as suggested by the authors. More appropriate and sensitive approaches, such as microdialysis, voltammetry, or electrophysiological recordings, are required to study extracellular glutamate dynamics.

 The presentation of the data is also very confusing. Group identities are indicated only by capital letters in all figures, without clear explanations. These group labels should be defined in the figures themselves or, at minimum, in the figure legends. The use of lowercase letters to denote statistical significance is similarly confusing. According to the figure legends, these letters appear to represent statistically significant differences between specific groups (e.g., b: comparison between the indicated group and the arsenic‑only group). However, this convention is not applied consistently. For instance, in Figure 2b, group F is marked with the letter b, despite showing xCT expression levels similar to those of group B. Moreover, the letter f, indicating p < 0.05 versus donepezil, appears in group I, which is the donepezil group itself.

 Another concern relates to the interpretation of the data. The effects of bromelain on glutamate receptor and transporter expression are described as strong. However, the levels of NR2A and EAAC1 following different doses of bromelain treatment remain significantly lower than control levels in both the hippocampus and prefrontal cortex (see Figs. 2a and 2c). Although the authors state that hippocampal NR2A expression in the medium‑dose group reached approximately 95% of control, this interpretation is not supported by the data presented in the histograms (compare bars A and G). Overall, these results do not support the conclusion that bromelain definitively reverses the suppressive effects of arsenic. Furthermore, medium and high doses of bromelain appear to significantly reduce glutamate levels compared with controls. While multiple types of glutamate transporters were examined, only a single NMDA receptor subunit was analyzed. Together, these findings may suggest that bromelain exerts some modulatory effects on glutamate homeostasis in the brain. However, describing these effects as preventing arsenic‑induced repression of essential excitatory signaling components or as restoring glutamate clearance and normal NMDAR function appears to be an overinterpretation of the data. The authors are therefore encouraged to tone down the implications of these findings.

 One issue raised in the previous round of review concerns confusion regarding sample size. This could be addressed by clearly reporting the n values in all figures.

 Finally, for the behavioral data, because escape latency was measured across multiple days of training as well as during the probe test, the authors should clarify which data were used to generate Figure 1B. Presenting escape latency data from both the training sessions and the probe test would allow a clearer assessment of the effects of bromelain on learning versus memory retrieval.

Is the work clearly and accurately presented and does it cite the current literature?

Yes

If applicable, is the statistical analysis and its interpretation appropriate?

Partly

Are all the source data underlying the results available to ensure full reproducibility?

Partly

Is the study design appropriate and is the work technically sound?

Partly

Are the conclusions drawn adequately supported by the results?

Partly

Are sufficient details of methods and analysis provided to allow replication by others?

Yes

Reviewer Expertise:

My research investigates how glutamate receptor function contributes to behavioral deficits in animal models of stress‑related psychiatric disorders.

I confirm that I have read this submission and believe that I have an appropriate level of expertise to confirm that it is of an acceptable scientific standard, however I have significant reservations, as outlined above.

# Reviewer report: Reviewer response for version 2

**DOI:** 10.5256/f1000research.194485.r446355

**Published:** 2026-01-10

**Authors:** Melissa Hernández-Frausto

**Affiliations:** 1 Neuroscience and Phisiology, New York University Langone Health, New York, New York, USA

The new version of the manuscript has been improved and most of my comments have been addressed with a precise and clear experimental design timeline figure and adding an Experimental grouping and treatment regimen in the main text. A very clear description of the Morris Water Maze test regarding the cues location has been addressed and the results title with the main finding is appreciated. As in my previous revision comment, a further analysis of the behavioral performance during the treatments such as latency to target, distance to find the target, velocity and quadrant position and trajectory would highly improve and strength the study. However, this new version with most of the comments addressed highly improves the study findings and clarification.

Is the work clearly and accurately presented and does it cite the current literature?

Partly

If applicable, is the statistical analysis and its interpretation appropriate?

Yes

Are all the source data underlying the results available to ensure full reproducibility?

Yes

Is the study design appropriate and is the work technically sound?

Yes

Are the conclusions drawn adequately supported by the results?

Partly

Are sufficient details of methods and analysis provided to allow replication by others?

Yes

Reviewer Expertise:

Neuroscience, Hippocampal function, Cortico-Hippocampal circuit interactions, Behavioral neurocience, Neurodegeneration

I confirm that I have read this submission and believe that I have an appropriate level of expertise to confirm that it is of an acceptable scientific standard.

# Reviewer report: Reviewer response for version 2

**DOI:** 10.5256/f1000research.194485.r446354

**Published:** 2026-01-08

**Authors:** Yousef Mohammed Aljohani

**Affiliations:** 1 Georgetown University Medical Center, Washington, Columbia, USA

The authors have adequately addressed my main concerns, particularly by clarifying the experimental timeline and explicitly acknowledging the staggered euthanasia of the arsenic-only group as a limitation. While additional behavioral and mechanistic depth would further strengthen the study, the revisions improve transparency and appropriately temper interpretation.

Is the work clearly and accurately presented and does it cite the current literature?

Yes

If applicable, is the statistical analysis and its interpretation appropriate?

Yes

Are all the source data underlying the results available to ensure full reproducibility?

Yes

Is the study design appropriate and is the work technically sound?

Partly

Are the conclusions drawn adequately supported by the results?

Partly

Are sufficient details of methods and analysis provided to allow replication by others?

Yes

Reviewer Expertise:

Neuropharmacology, Aging Neuroscience, Catecholamine Neurotransmission, Glutamatergic Regulation, and Synaptic Plasticity

I confirm that I have read this submission and believe that I have an appropriate level of expertise to confirm that it is of an acceptable scientific standard.

# Reviewer report: Reviewer response for version 1

**DOI:** 10.5256/f1000research.186610.r424625

**Published:** 2025-11-11

**Authors:** Melissa Hernández-Frausto

**Affiliations:** 1 Neuroscience and Phisiology, New York University Langone Health, New York, New York, USA

The article starts with a detailed description on how excitatory neurotransmission associated to NMDAR receptors and transporters are highly involved in neurological disorders. How arsenic exposure cause neurotoxicity and cognitive function triggering a range of detrimental mechanisms and how phytochemical compounds specifically, bromelain can have a protective effect to restore neurological function leading to an improvement in cognitive performance.  

 Here, Anyanwu et al., in seventy-two male rats randomly divided into nine groups with arsenic treatment only (20 mg/kg/day) for 14 days, bromelain-only (5-15 mg/kg/day), arsenic+bromelain ( 5-15 mg/kg/day) and arsenic+donepezil (2 mg/kg/day) for 14 days (28 days in total) were evaluated first in the Morris water maze to assess hippocampal related cognitive performance. Then brains were extracted, and glutamate concentration was measured, as well as expression of RNA from NR2A, GLT-1, EAAC1 and xCT. They found that arsenic by itself, impaired spatial memory, increased glutamate levels, and decrease expression of NR2A and transporter genes. Interestingly, treatment with bromelain at 10 mg/kg dose was capable to reestablished gene expression and lowered glutamate levels, whereas bromelain at 15 mg/kg, the highest dose was more effective than donepezil in improving glutamate clearance and cognitive performance.

 The article is a relevant study on topics related to arsenic neurotoxicity and bromelain in cognitive and glutamatergic system improvement with a highly number of groups assessing the different doses of bromelain and arsenic and a positive control as the donepezil. Very clearly explained the methods with a detailed explanation of ethical approval, experimental animals and housing, sample size and power calculation, experimental grouping and treatment regimen, animal monitoring and welfare, experimental timeline, and the behavioral and tissue collection and processing. However, I have some comments and concerns about the article’s structure that I believed can highly improve the manuscript.

  

1. As there are a high number of experimental groups on the Experimental grouping and treatment regimen, an experimental design figure should be made so it’s clearer for the readers the groups analyzed at the behavioral and molecular performance. This will help to clarify if the arsenic + bromelain treatment with the different doses was in co-treatment or not and the timing of behavioral and brain extraction.
2. Why only used males at 8 weeks of old? Is there difference between males and females in arsenic neurotoxicity? Is there difference between adolescent or adult rats? Please justify and specify what is the rationale of this.
3. In the Morris Water Maze Test description, specify if there are any global cues located around the maze. Are the cues located in a determined distance and maintained during the whole behavioral performance? Please clarify and write in behavioral assessment.
4. All results should start with the main finding. As example: Instead of Behavioral performance in Morris Water Maze, it should state " Bromelain restored cognitive performance in animals exposed to arsenic". Like the paragraph at the beginning of the graph that it mentions the main result or as the title of the figure legends. This with all the results from the figures.
5. For the behavioral performance in Morris Water Maze. More analysis will highly improve the quality of the findings, besides the only analysis of latency. They should divide the analysis between acquisition and retrieval and analyze for each one the latency to target, distance to find the target, velocity, the quadrant position and trajectory to see if the effect of bromelain improve only some or all the parameters.
6. In results, all the findings should be described accordingly to the group but also to the figure and panels to guide us as readers where is the finding that is being described.
7. The figures should be highly improved. Change the figures for a scatter plot with bar or box plot with individual animals, this will highly impact the results visualization and the quality of the figures. The significance should be addressed as * instead of letters and align in the figure which groups are different among them. As now, the results are very confusing and not clear to see the differences.
8. There is no consistency among the groups, in the methodology it states that for statistical power an n=8 was used whereas in the RNA expression and in discussion it mentioned an n=5. Please clarify and justify the differences written.
9. Introduction will highly improve if first states with the Arsenic neurotoxicity, then the link to glutamate signaling and finally the bromelain as an improvement mechanism.

Minor comments:

- **In Page 4:** Its Alzheimer’s disease not Alzheimer. This phrase “ *Arsenic, at the cellular level, arsenic triggers a range of detrimental mechanisms, such as oxidative stress, mitochondrial dysfunction” *is not clear and is not well written. This phrase “ *it is either there* *is an excess of extracellular glutamate and, consequently, an overactivation of NMDARs, resulting in impairment of synaptic plasticity and death of neurons.”* It is either there is not correct, improve the grammar.
- **In Experimental animals and housing conditions: **Specify if the light/dark cycle is inverted. This phrase “ *The acclimatization period to the facility was 14 days after the start of the experiment.” *Clarify if the acclimatization to period was after the start of the experiment or before.

Is the work clearly and accurately presented and does it cite the current literature?

Partly

If applicable, is the statistical analysis and its interpretation appropriate?

Yes

Are all the source data underlying the results available to ensure full reproducibility?

Yes

Is the study design appropriate and is the work technically sound?

Yes

Are the conclusions drawn adequately supported by the results?

Partly

Are sufficient details of methods and analysis provided to allow replication by others?

Yes

Reviewer Expertise:

Neuroscience, hippocampal function, Behavioral neurocience, neurodegeneration

I confirm that I have read this submission and believe that I have an appropriate level of expertise to confirm that it is of an acceptable scientific standard, however I have significant reservations, as outlined above.

# Response

**Published:** 2025-12-27

**Authors:** Emeka Anyanwu

**Affiliations:** Anatomy, Biomedical Sci., Kampala International University Faculty of Medicine and Dentistry, Kampala, Central Region, Uganda

**Reviewer 2**

 • **Comment 1 (Methodology & experimental design; design clarity/timeline confound):** *Clarify co-treatment vs post-treatment; arsenic-only euthanized earlier (day 15) vs others day 28; acknowledge confound/recovery*

 Thanks so much for this insight. We have provided an additional  figure illustrating  specifically that arsenic was given 14 days and bromelain/donepezil was given 15-28 (post-exposure treatment) and that the arsenic-only group had been euthanized earlier (day 15) and that other groups were euthanized day 28. We shall also revise the inconsistent phrasing in the abstract (coadministered) that now distorts the regimen presented in the Methods.

 We acknowledge the **staggered euthanasia** (Group B at day 15; others at day 28) as a limitation that may confound treatment effects with time-related recovery , and we will add a “future work” note recommending an arsenic→vehicle recovery control group as suggested.

 • **Comment 2 (Neuroscience interpretation; protein validation):** *Claims rely only on mRNA; avoid overstating; add protein-level validation or tone down*.

**Response**: We agree with you. We have explicitly  mentioned the absence of protein validation as a study limitation; we further reinforced this point by (i) being less mechanistic in the Results/Discussion, and (ii) making changes in our Conclusion  

  • **Comment 3 (Methodology; glutamate assay interpretation):** *ELISA measures total tissue glutamate, not extracellular levels; interpret accordingly.*

**Response:** Agreed. We have amended the discussion to make it clear that the competitive ELISA is a measure of total tissue glutamate in the homogenate and not a measure of synaptic/extracellular glutamate dynamics that are of interest in excitotoxicity. We have adjusted mechanistic inferences to this extent and reported glutamate findings as an indirect measure in line with modified glutamate processing.

 • **Comment 4 (Reporting/reproducibility; sample size inconsistency):** *Methods say n=8 but gene expression uses n=5; report exclusions/attrition*

 Response: Thanks for pointing our attention to this ambiguity, we have clarified this in our  revision.   The power/sample-size n now has n=8 per group but qPCR gives n=5 and subsequently in limitations captures small n. In the revision we have:

- Clearly specified which endpoints employed n=8 vs n=5 (e.g., behavior vs molecular/biochemical subsets).
- Made sure that the proper n of each dataset is reported in figure legends as well as in the Statistical Analysis section.

• **Comment 5 (Statistical rigor; multiple testing):** *Multiple ANOVAs across genes/regions need correction for multiple comparisons.*

**Response:** Thank you. Although the manuscript mentions the use of Bonferonni correction regarding the repeated-measures learning-curve comparison, we have included an explicit plan of multiplicity control across the molecular endpoints (i.e., Benjamini-Hochberg FDR across the gene panel in each region, and/or across regions) and have provided adjusted p-values and raw p-values.  

  Conclusions.

 • **Comment 6 (Neuroscience-specific interpretation; mechanistic speculation):** *NF-κB/Nrf2/ERK/BDNF are hypothetical; clearly label as untested*

**Response:** We agree. These pathways have been proposed in the Discussion as hypotheses; we  have made it clear   that they are speculative and untested in this study . We have also ensured that the Conclusions do not suggest pathway validation.

 • **Minor 1 (Literature currency):** *Cite primary studies on bromelain BBB permeability*

 Response: Done. Thanks

 • **Minor 2 (Figures/reporting):** *Figures lack y-axis units (MWM) and individual data points; add scatter or box plots to display variance.*

**Response:** Well noted for implementation in the revision

 • **Minor 3 (Terminology/typos):** *Use EAAC1/EAAT3 consistently; replace “arsenic brains.”*

 Response: Thanks so much for calling our attention to this. We have deleted EAAT3 from the manuscript using only **EAAC1 **to ensure consistence as advised.  

*“arsenic brains.”* Has been changed to “arsenic-exposed brains” in keeping with your  suggestion.

 • **Minor 4 (Stats reporting):** *List F-values and degrees of freedom for ANOVA in figure legends.*

**Response:** Noted for implementation in the revised manuscript.

 Line-specific notes:

**Response:**

1. The correction of the acclimatization timing will be done to the time before the beginning of the experiment.
2. The unit of humidity has been corrected from   50-60 ^0^C  to  50-60%.
3. The MWM has been be expanded with cue description and intervals.
4.  Revised version now has supernatant was “ **collected**”, which is consistent with the planned procedure.
5. sample size discrepancy (n=8 vs n=5) has been addressed above
6. Claims that bromelain has been shown to be better than donepezil has been toned down,  the Results already indicates a non-significant behavioral difference (15% less, but non-significant) and we will make sure Abstract/Conclusion does not overstate the superiority argument.
7. To avoid ambiguity, the comparison made with NR2B has been deleted,  to keep the sentence simple
8. We have included the  statement: “that tissue glutamate does not measure extracellular pools”.

We are really grateful for the improvement you added to the work. Thank you

# Reviewer report: Reviewer response for version 1

**DOI:** 10.5256/f1000research.186610.r421876

**Published:** 2025-10-17

**Authors:** Yousef Mohammed Aljohani

**Affiliations:** 1 Georgetown University Medical Center, Washington, Columbia, USA

Summary:

 The authors test the hypothesis that bromelain reverses arsenic-induced excitotoxicity by restoring glutamatergic signaling. Seventy-two rats were divided into nine groups receiving sodium arsenate, bromelain (5–15 mg/kg), or donepezil. They report that arsenic increased brain glutamate and downregulated NR2A, GLT-1, EAAC1, and xCT mRNA, whereas bromelain normalized these gene expressions and improved Morris Water Maze performance. The topic is timely, linking environmental neurotoxicity and natural neuroprotectants, but several methodological and interpretative issues need to be addressed.  

 2. Major strengths

1. Ethical and statistical transparency: explicit IACUC approval and a priori G*Power sample-size calculation are commendable.
2. Appropriate behavioral and molecular endpoints: MWM and qPCR for transporter genes are relevant to glutamatergic homeostasis.
3. Dose–response exploration: inclusion of three bromelain doses (5–15 mg/kg) and a donepezil comparator provides valuable context for efficacy.
4. Clear reporting of control groups and blinding: the manuscript notes observer blinding during behavioral testing.

3. Major concerns

1. Design clarity and timing: arsenic exposure was followed by bromelain for 14 days, yet the abstract and discussion describe co-treatment. Clarify whether bromelain prevents or reverses toxicity, and whether rats were on arsenic during the bromelain treatment or not. Importantly, the timeline design introduces a minor confound: the arsenic-only group (Group B)  was euthanized earlier (day 15) than treatment groups (day 28), which could conflate treatment effects with spontaneous recovery. Future studies could include a delayed “arsenic → water” recovery group or acknowledge this as a timeline limitation to strengthen causal inference.
2. Lack of protein-level validation: mechanistic claims rest solely on mRNA data without Western blot or immunohistochemistry confirmation. It was clarified in the discussion, but the conclusion should not be overstated.
3. Glutamate assay limitations: ELISA measures total tissue glutamate, not extracellular levels relevant to excitotoxicity that can be done with microanalysis or other technique; interpret accordingly.
4. Sample size inconsistency: methods state n=8 per group, but gene expression used n=5; justify attrition and report exclusion criteria. Two parts contradict the justification for the sample size in the method.
5. Statistical rigor: multiple ANOVAs across genes and regions lack correction for multiple testing; consider FDR or Bonferroni adjustment.
6. Mechanistic speculation: references to NF-κB, Nrf2, and ERK/BDNF signaling are hypothetical; clearly identify as untested.

4. Minor and technical issues

1. The introduction could cite primary mechanistic studies on bromelain’s blood–brain barrier permeability.
2. Figures lack y-axis units (MWM) and individual data points; add scatter or box plots to display variance.
3. Typographical corrections: use 'EAAC1/EAAT3' consistently and replace 'arsenic brains' with 'arsenic-exposed brains.'
4. List F-values and degrees of freedom for ANOVA in figure legends.

5. Overall assessment

 This study provides preliminary evidence that bromelain mitigates arsenic-induced cognitive and molecular deficits. The findings are plausible but not definitive, as the evidence is confined to mRNA, glutamate measures are indirect, and mechanistic claims exceed the available data. With clarification of treatment timing, protein-level validation, and tempered interpretation, the manuscript would be suitable for publication.

 6. Line-specific editorial notes

1. Page 5: Correct acclimatization timing ('before', not 'after').
2. Page 5: Correct humidity units ('50–60 °C' → '50–60%').
3. Page 6: Add details on MWM (visual cues, inter-trial interval, locomotion).
4. Page 8: Clarify sample size discrepancy (n=8 vs n=5). "Hippocampal tissue samples (~50 mg) were homogenized in ice-cold 0.1 M phosphate-buffered saline (pH 7.4) and centrifuged (10,000 g, 10 min, 4°C) to obtain supernatants. The supernatants were discarded, and the samples were diluted and subjected to ELISA." I believe you mean here to collect the supernatant? 
5. Pages 9–11: Avoid stating bromelain 'outperformed donepezil' unless statistically validated.
6. Pages 12–13: Include NR2B  consideration and add protein validation (Western/IHC).
7. Page 14: Note that the total glutamate ELISA does not measure extracellular pools.

7. Recommendation and competing interests

 The study is promising but needs improved methodological transparency, data validation, and conservative interpretation. Once revised, it could contribute meaningfully to research on environmental neurotoxicity and glutamatergic signaling.

Is the work clearly and accurately presented and does it cite the current literature?

Yes

If applicable, is the statistical analysis and its interpretation appropriate?

Yes

Are all the source data underlying the results available to ensure full reproducibility?

Yes

Is the study design appropriate and is the work technically sound?

Partly

Are the conclusions drawn adequately supported by the results?

Partly

Are sufficient details of methods and analysis provided to allow replication by others?

Yes

Reviewer Expertise:

Neuropharmacology, Aging Neuroscience, Catecholamine Neurotransmission, Glutamatergic Regulation, and Synaptic Plasticity

I confirm that I have read this submission and believe that I have an appropriate level of expertise to confirm that it is of an acceptable scientific standard, however I have significant reservations, as outlined above.

# Response

**Published:** 2025-12-27

**Authors:** Emeka Anyanwu

**Affiliations:** Anatomy, Biomedical Sci., Kampala International University Faculty of Medicine and Dentistry, Kampala, Central Region, Uganda

**Reviewer 1**

**Comment 1 (Figures/tables/supplement; design clarity):** *Add an experimental design figure; clarify whether arsenic+bromelain is co-treatment and timing of behavioral tests/tissue collection*

 Response: Thank you, this is important with regard to transparency.. We have included a new Figure which will depict the required clarifications starting from-(i) acclimatization, (ii) arsenic exposure phase, (iii) post-exposure treatment phase (bromelain/donepezil), (iv) MWM acquisition/probe timing, and (v) euthanasia/tissue collection

 This figure illustration specified that arsenic was given 14 days and bromelain/donepezil was given 15-28 (post-exposure treatment) and that the arsenic-only group had been euthanized earlier (day 15) and that other groups were euthanized day 28. We shall also revise the inconsistent phrasing in the abstract (coadministered) that now distorts the regimen presented in the Methods.

**Comment 2 (Methodology & experimental design; sex/age rationale):** *Why only 8-week-old males? justify sex/age; address potential sex differences*

**Response**: We have included justification in **Experimental animals and housing conditions **section of the manuscript, stating why 8-week-old male Wistar rats were chosen ( and this is- to reduce the variation in behavioral results due to hormonal cycles and to align with commonly used for arsenic neurotoxicity model systems), and we will explicitly note **sex as a biological variable limitation** and recommend that females be included in future studies. This limitation will also be captured in the Discussion and Conclusions.

**Comment 3 (Methodology; MWM reporting/reproducibility):** *Specify global cues around the maze; whether cues remained fixed.*

**Response**: We revised the method part of MWM to include more details on the cues ensuring our method is replicable.

**Comment 4 (Writing clarity/structure):** *Results headings should start with the main finding; strengthen figure legends similarly.*

**Response:**  We quite agree with you and appreciate the suggestion. We have ensured that all the titles of Results, subsection and figure legends  emphasize  the main finding.

**Comment 5 (Statistical analysis & rigor; behavioral analytics):** *MWM analysis should go beyond latency; separate acquisition vs retrieval; include distance, speed, quadrant time/trajectory*

 Response: Thank you. During the experimental part of this work, our major focus was on the latency. We would have included more parameters as suggested if it were possible. We have separated  acquisition and retrieval stages in our revision.

**Comment 6 (Writing clarity; results navigation):** *Describe findings with group, figure, and panel references.*

 Response:  Thanks. Well noted.  

**Comment 7 (Figures/tables):** Improve figures—scatter/box with individual points; use * for significance not letters; improve clarity.

**Response**: We really agree with you that using  * for significance makes for better clarity   than letters, currently all our figure have letter symbols, but  we have  ensured their meanings are well specified. We will stick to special symbols as *  in subsequent research as you have noted. thanks.

**Comment 8 (Data reporting/reproducibility):** *n=8 in methods vs n=5 in RNA/discussion—clarify and justify*

 Response: Thanks for pointing our attention to this ambiguity.  In the revision we have :

- Clearly specified which endpoints employed n=8 vs n=5 (e.g., behavior vs molecular/biochemical subsets).
- We made sure that the proper n of each dataset is reported in figure legends as well as in the Statistical Analysis section.

• **Comment 9 (Conceptual framing):** *Introduction: start with arsenic neurotoxicity → glutamate signaling → bromelain.*

**Response**:  Thanks for this useful suggestion, we also appreciate its appropriateness. We also acknowledge that the experimental driver of the model is arsenic neurotoxicity; but we purposefully started the Introduction with glutamatergic signaling, transporter/NMDAR biology since it is the main mechanistic target of this study (NR2A, GLT-1, EAAC1, xCT) and the core of the hypothesis. Instead of the core conceptual theme, arsenic is then added as a clinically relevant perturbation of glutamatergic homeostasis. This framework also allows generalisability outside the field of arsenic toxicology, and retains a consistent flow of the supportive body of literature, as already referenced. In response to the concern of the reviewer, we have enhanced the switch to arsenic by conspicuously indicating that the impairment of cognition by arsenic is largely by disrupting glutamate homeostasis, the basis behind testing of bromelain as a glutamatergic modulator..  

**Minor remarks (Writing clarity/typos):**

 Response: We have made all the correction as suggested.  

 Thanks so much for the improvements added to the work. We are very grateful
